# Can rumen bacteria communicate to each other?

**DOI:** 10.1186/s40168-020-00796-y

**Published:** 2020-02-21

**Authors:** Mi-Young Won, Linda B. Oyama, Stephen J. Courtney, Christopher J. Creevey, Sharon A. Huws

**Affiliations:** 1 Institute of Biological, Environmental and Rural Sciences (IBERS), Aberystwyth University, Penglais Campus, Aberystwyth, SY23 3DA UK; 2 School of Biological Sciences, Institute for Global Food Security, Queen’s University of Belfast, 19 Chlorine Gardens, Belfast BT9 5DL, Northern Ireland, UK

**Keywords:** Rumen, Quorum sensing, Bacteria, Acyl-homoserine lactone, LuxS, AI-2

## Abstract

**Background:**

The rumen contains a myriad of microbes whose primary role is to degrade and ferment dietary nutrients, which then provide the host with energy and nutrients. Rumen microbes commonly attach to ingested plant materials and form biofilms for effective plant degradation. Quorum sensing (QS) is a well-recognised form of bacterial communication in most biofilm communities, with homoserine lactone (AHL)-based QS commonly being used by Gram-negative bacteria alone and AI-2 Lux-based QS communication being used to communicate across Gram-negative and Gram-positive bacteria. However, bacterial cell to cell communication in the rumen is poorly understood. In this study, rumen bacterial genomes from the Hungate collection and Genbank were prospected for QS-related genes. To check that the discovered QS genes are actually expressed in the rumen, we investigated expression levels in rumen metatranscriptome datasets.

**Results:**

A total of 448 rumen bacterial genomes from the Hungate collection and Genbank, comprised of 311 Gram-positive, 136 Gram-negative and 1 Gram stain variable bacterium, were analysed. Abundance and distribution of AHL and AI-2 signalling genes showed that only one species (*Citrobacter* sp. NLAE-zl-C269) of a Gram-negative bacteria appeared to possess an AHL synthase gene, while the Lux-based genes (AI-2 QS) were identified in both Gram-positive and Gram-positive bacteria (191 genomes representing 38.2% of total genomes). Of these 192 genomes, 139 are from Gram-positive bactreetteria and 53 from Gram-negative bacteria. We also found that the genera *Butyrivibrio*, *Prevotella*, *Ruminococcus* and *Pseudobutyrivibrio*, which are well known as the most abundant bacterial genera in the rumen, possessed the most lux-based AI-2 QS genes. Gene expression levels within the metatranscriptome dataset showed that *Prevotella*, in particular, expressed high levels of LuxS synthase suggesting that this genus plays an important role in QS within the rumen.

**Conclusion:**

This is the most comprehensive study of QS in the rumen microbiome to date. This study shows that AI-2-based QS is rife in the rumen. These results allow a greater understanding on plant-microbe interactions in the rumen.

## Background

The rumen microbiome is a highly specialised, complex community composed of bacteria, protozoa, fungi and viruses [1]. Even though the rumen microbiome has been studied for many years, progress in understanding rumen microbial function has been slow due to the complexity of the ecosystem and available technologies, although recent ‘omic advances have improved our understanding. Despite these recent developments of ‘omic technologies, there remains to be a dearth of knowledge regarding bacterial cell to cell communication in the rumen microbiome. Much is known about cell communication from pure culture studies of non-ruminant origin (e.g. *Vibrio harveyi*, *V. fischeri*, *Escherichia coli*, *Pseudomonas aeruginosa*), which shows that Gram-negative bacteria typically use acylated homoserine lactone (AHLs)-based quorum sensing (QS) cell to cell communication strategies (autoinducer-1 QS system; AI-1) and Gram-positive bacteria use furanosyl borate diester or tetrahydroxy furan (autoinducer-2 QS system; AI-2) [2–4]. In these bacteria, upregulation of QS autoinducer chemicals, followed by receptor binding, instigates changes in overall bacterial gene expression and phenotype, with increased virulence commonly being a consequence [4]. These studies outline the genetic basis of QS-based bacterial cell to cell communication in vitro and are useful for furthering our understanding; however, their applicability to microbiome communities are uncertain and cell to cell communication in multi-species biofilms, such as those in the rumen, are likely to be far more complicated.

AHLs have been detected in rumen fluid, suggesting they may play a role in cell to cell communication in the rumen microbiome [5]. By testing pure cultures, Erikson et al. (2002) [5] were not able to identify which bacteria produce AHL using Gram-negative rumen bacterial pure cultures, including *Anaerovibrio lipolyticus* 5S, *Fibrobacter succinogenes* S85, *Megasphaera elsdenii* LC1, *Prevotella albensis* 223/M2/7, *P. brevis* GA33, *P. bryantii* B14, *P. ruminocola* 23 and B85, *Ruminobacter amylophilus* 70 and WP109, *Selenomonas ruminantium* HD4, four unnamed *S. ruminantium* strains and *Succinovibrio dextrinosolvens* 24, as well as Gram-positive ruminal pure culture bacteria *Butyrivibrio fibrisolvens*, *Lachnospira multiparus* 20, *Ruminococcus albus* B199, two strains of *R. flavefaciens* and *Streptococcus bovis* YM150. This suggests that the as yet unculturable bacteria or other cultured bacteria not tested by Erickson et al. (2002) [5] may be responsible for most of the AHL signals found in rumen fluid. Interestingly, it has been reported that AHL signals are reduced in concentration in rumen fluid taken during the winter, possibly due to changes in the microbiome in line with winter diets [6]. While recently, Yang and colleagues isolated *P. aeruginosa* YZ_1_ from the rumen of cattle, which utilises AHL signals to communicate [7], although this bacterial genus is not commonly found in the rumen ecosystem [8]. Supporting the importance of cell to cell communication in the rumen microbiome, LuxS proteins and analogues from the AI-2 QS system have been annotated in transcriptome datasets from the rumen [9]. Moreover, AI-2 activity was also detected in the rumen contents of three cows [10–12]. Mitsumori and colleagues (2003) [10] also detected AI-2 signals in pure cultures of *B. fibrisolvens*, *Eubacterium ruminantium*, *Ruminococcus flavefaciens* and *Succinomonas amylolytica*, suggesting a prominent role of AI-2-based QS in the rumen.

Recently, the Hungate collection of rumen microbial genomes was released [13]. This collection of 501 microbial genomes is the most comprehensive for the rumen microbiome and represents a major step change in our ability to understand the rumen microbiome. In this study, we took advantage of the availability of the Hungate collection, alongside other rumen bacterial genomes deposited in Genbank, to prospect rumen bacterial genomes for genes/proteins which are known to be involved in QS cell to cell communication and to further our understanding of the importance of QS in the rumen. We also confirmed that putative QS genes were expressed by prospecting for these sequences in the largest available rumen metatranscriptome datasets.

## Methods

### Bacterial genomes used in this study

The bacterial genomes used in this study were obtained both from the Hungate collection (428 bacterial species excluding genomes from archaea, viruses and eukaryotes) (https://genome.jgi.doe.gov/portal/HungateCollection/HungateColl-ection.info.html) (accessed on July 2018) and from Genbank (https://www.ncbi.nlm.nih.gov/genome) (July 2018) [13]. To find the genomes in Genbank, the genomes category in PubMed was searched using the keywords “rumen and bacteria”. Only Genbank results from complete genomes not represented in the Hungate collection were used in this study. The Genbank search produced 20 rumen bacteria genomes, 12 Gram-positive and 8 Gram-negative species. Combined with the 428 bacterial genomes from the Hungate collection, this resulted in genomes from a total of 448 rumen bacteria species, 311 which were Gram-positive, 136 which were Gram-negative and 1 which was Gram stain variable (Additional file 1: Table S1). These were then analysed for genes involved in QS cell to cell communication.

### Prospecting genomes for quorum sensing-related proteins

Each of the genomes was re-annotated using PROKKA (using default settings), which uses BLAST+ and Blastp to annotate, and for each genome, the resulting annotations of the predicted protein sequences were searched using semantic approaches [14]. To search for AHL-related genes, the following search terms were prospected: Quorum sensing, autoinducer, AHL, HSL, homoserine lactone, N-acyl homoserine, AHL synthase, RhlI, LuxI, LuxR, LasI, LasR and homoserine lactone efflux protein. To search for AI-2-related genes, the following search terms were prospected: Quorum sensing, autoinducer, LuxS, LuxP, LuxQ, S-ribosylhomocysteine, S-ribosylhomocysteine lyase (full chemical name for Lux S) and AI-2.

### Sequence alignment and generation of phylogenetic trees

All LuxS proteins identified from the rumen bacteria were aligned using Mega7 (v. 7.0.26) [15]. A phylogenetic tree was constructed with additional protein genes from *Vibrio harveyi* (Gram-negative) and *Streptococcus pneumoniae* (Gram-positive), in which LuxS based on QS system have been well studied, alongside LuxS-based QS proteins previously identified from rumen microbial metagenomics and metatranscriptomic sequences [9], for comparative purposes. Phylogenetic trees were constructed using neighbour-joining clustering method [16]. A bootstrap consensus tree was inferred from 1000 replicates, and branches corresponding to partitions reproduced in less than 50% bootstrap replicates are collapsed. The evolutionary distances were computed using the Poisson correction method and are represented by in number of amino acid substitutions per site. The analysis involved 201 amino acid sequences. All ambiguous positions were removed for each sequence pair. There were a total of 191 positions in the final dataset.

### LuxS synthase expression within a rumen bacterial metatranscriptome

Twenty publicly available metatranscriptomic datasets were taken from the National Center for Biotechnology Information Sequence Read Archive, under the accession number SRA075938 [16]. The samples were 150 bp paired-end reads from the Illumina Hiseq2000 sequencer [16]. Fastq files were processed with multiqc [17], and reads were trimmed from 150 to 110 bp using the Trimmomatic software version 0.36 [18]. Reads were aligned to the Hungate rumen genome dataset using bowtie2 version 2.3.0 [19] using the settings “--very-sensitive-local” which allowed soft trimming of the reads and a relaxed alignment and “-k 497”. The resulting SAM files were converted to BAM files using SAMtools version 1.9 [20]. SAMtools version 1.9 was used to filter all and the best alignment position for each read using the flag option “-F 260”. For each of the 20 final filtered BAM files FeatureCounts (from the subread package version 2.0.0) [21] was used to calculate the number of reads that align within the boundaries of every predicted gene in the Hungate genomes. The read counts were then converted into RPKM values. Finally, the RPKM values of the genes of interest in this study were extracted from the entire expression count table.

## Results

### Quorum sensing-related proteins with rumen bacterial genomes

For the purpose of analysis, two genera, *Lachnobacterium* and *Micrococcus*, which are often described as weakly Gram-positive or Gram-variable respectively were included with the Gram-positive bacteria group (Additional file 1: Table S1). *Flavonifractor plautii*, which is also a Gram-variable bacterium, but never referred to as being weakly Gram-positive or negative, was consequently not included in either group (Additional file 1: Table S1). Fifty percent of the genome sequences prospected for QS cell to cell signalling did not contain any protein annotated with LuxS or AHL signalling system, irrespective of their Gram staining phenotypes. Thus, any genomes which did not have LuxS or AHL signalling genes were presented as N/A (not applicable; Additional file 1: Table S1). When specific species names or strain ID could not be identified from Genbank genome entries, results were included with closely related genera or families in the Hungate collection (e.g. family: *Crodiobacteriaceae*, genus: *Olsenella* and family: *Propionibacteriaceae*, genus: *Propionibacterium*). This was also done when species had been re-classified with different names, e.g. *Clostridium mangenotii* and *Eubacterium rectalis* have been re-classified as *Clostridioides mangenotii* and *Agathobacter rectalis* respectively.

**Fig. 1:**
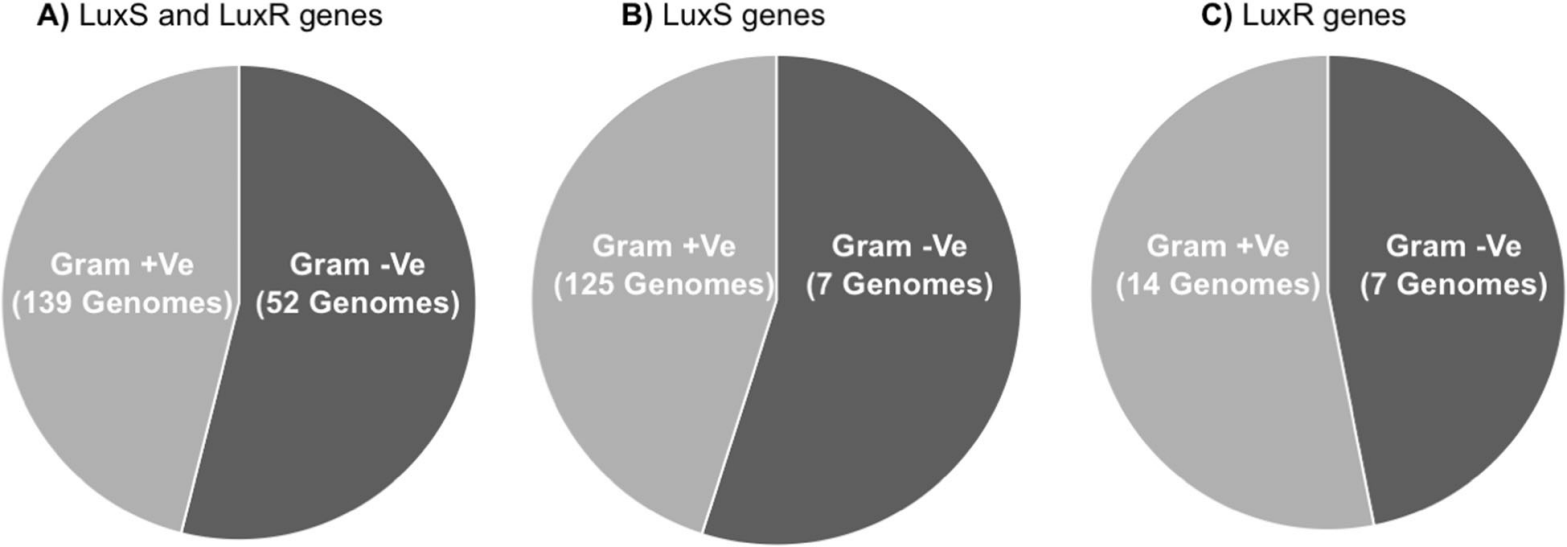
Percentage occurrence of putative AI-2 LuxS-based quorum sensing proteins in the genomes of Gram-positive and Gram-negative rumen bacteria [13]. **a** Proportional representation of LuxS and LuxR in the genomes of the Gram-positive and Gram-negative genomes. **b** Proportional representation of LuxS in the genomes of the Gram-positive and Gram-negative genomes. **c** Proportional representation of LuxR in the genomes of the Gram-positive and Gram-negative genomes. Numbers in brackets show total number of bacterial genomes in which the corresponding gene was found

LuxS proteins, which can produce/regulate AI-2 signal and the corresponding receptor protein LuxR, were highly represented in both Gram-positive and Gram-negative bacteria species. A total of 171 LuxS proteins were identified in the genomes analysed, with 40.2% and 33.0% of LuxS proteins being found within Gram-positive and Gram-negative bacteria species respectively (Fig. 1; Additional file 1: Table S1). A total of 21 LuxR proteins were also identified in the genomes analysed, with 4.5% and 5.1% of LuxR proteins being found within Gram-positive and Gram-negative bacteria species respectively (Fig. 1; Additional file 1: Table S1). In contrast, only one species (*Citrobacter* sp. NLAE-zl-C269) appeared to have the AHL synthase gene among the Gram-negative bacteria list (Additional file 1: Table S1). Nonetheless, AHL efflux proteins were detected in six (*Acinetobacter*, *Enterobacter*, Porphyromonadaceae, *Prevotella*, *Oxalobacter*, *Wolinella*) Gram-negative bacterial genomes (4.4%), with no AHL-related proteins being detected in Gram-positive bacteria as expected. *Citrobacter* sp. NLAE-zl-C269 also possessed luxS and LuxR genes, suggesting that this bacterium may use the AHL and AI-2 quorum sensing systems (Fig. 1; Additional file 1: Table S1). On further note, nine of the rumen bacterial genomes (Bacillus sp. MB2021, *Blautia schinkii* DSM 10518, *Dorea longicatena* AGR2136, Lachnospiraceae bacterium FE2018, *Slackia heliotrinireducens*, *Oscillibacter* sp. PC13, *Oxalobacter formigenes*, *Sagittula stellata*, Selenemonas ruminantium) also contained AHL lactonase genes which are used to cleave the lactone ring. This suggests that these rumen bacteria may have the ability to reduce AHL-based QS in the rumen through degradation of AHL.

**Fig. 2:**
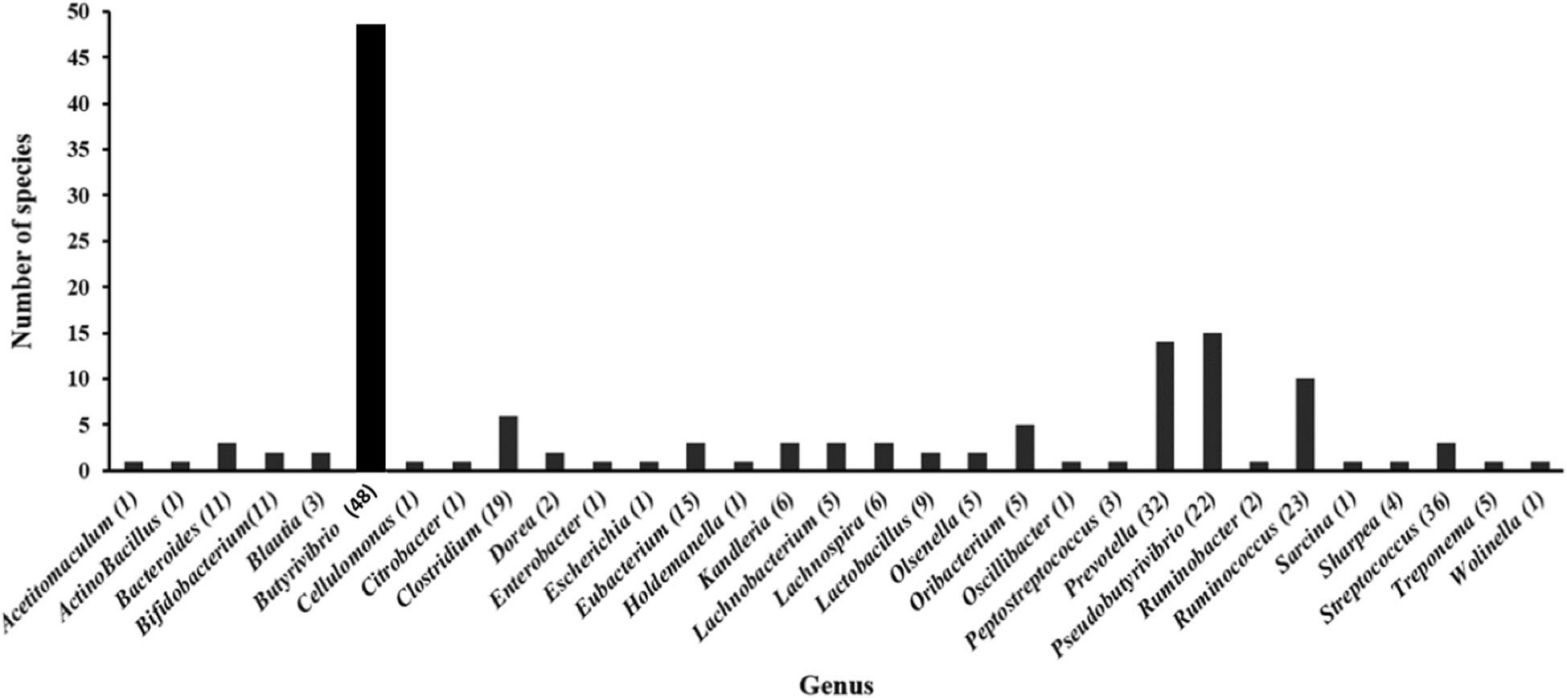
Distribution and abundance of LuxS proteins relating to AI-2-based quorum sensing across 448 rumen bacterial genomes [13]

**Fig. 3:**
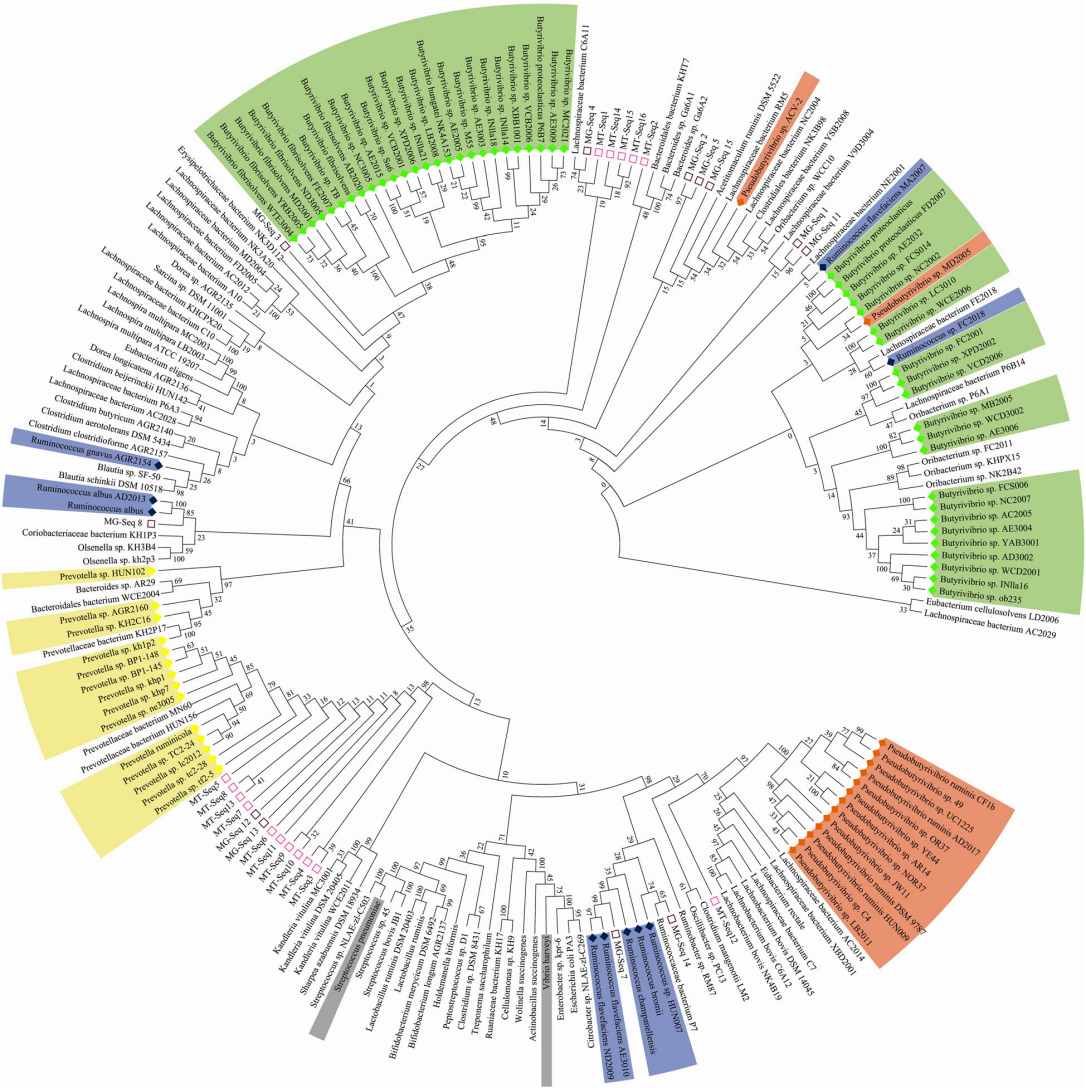
Comparative phylogenetic tree of all putative LuxS proteins detected within the genomes of 171 rumen bacterial species. Two standards (*Vibrio harveyi* and *Streptococcus pneumoniae*) were used for comparison with other samples (highlighted with grey colour). Most abundant four groups were highlighted with colours (orange: *Pseudobutyrivibrio*, yellow: *Prevotella*, green: *Butyrivibrio* and blue: *Ruminococcus*). Sequences denoted by a square and beginning with MG are those identified by Ghali et al. [9] from rumen metagenomic sequences and those with MT also being derived from the same study using a metatranscriptome dataset

**Fig. 4:**
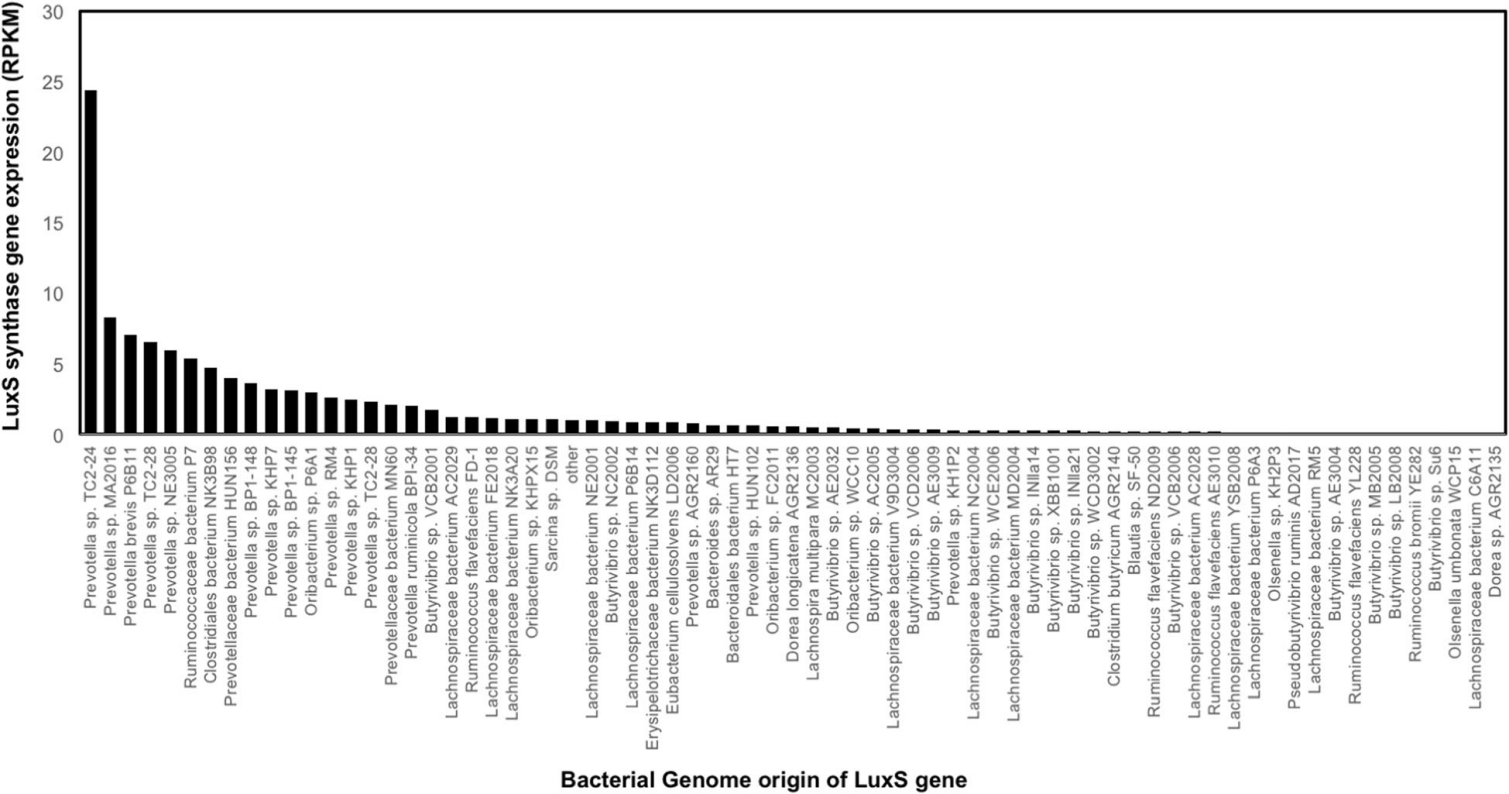
Average expression of LuxS genes identified in the bacterial genomes within rumen bacterial metatranscriptome datasets [16]. Expression is shown as reads per kilobase of transcript per million (RKPM). Where RKPM was < 0.1, the expression data was grouped as “other”. Other essentially contained the expression of the LuxS synthase genes discovered within *Lachnobacterium bovis* DSM, *Ruminococcus gnavus* AGR2154, *Actinobacillus succinogenes* 130Z, *Streptococcus* sp. NLAE-zl-C503, *Butyrivibrio* sp. AD3002, *Butyrivibrio* sp. INlla18, *Pseudobutyrivibrio ruminis* DSM, *Bifidobacterium bifidum* Calf96, *Butyrivibrio* sp. TB, *Butyrivibrio* sp. WCD2001, *Succinimonas_amylolytica* DSM 2873, *Pseudobutyrivibrio* sp. JW11, *Ruminococcus flavefaciens* 17, *Pseudobutyrivibrio* sp. LB2011, *Ruminococcus albus* SY3, *Pseudobutyrivibrio* sp. MD2005, *Butyrivibrio* sp. NC3005, *Clostridium mangenotii* LM2, *Cellulomonas* sp. KH9, *Clostridium aerotolerans* DSM, *Butyrivibrio fibrisolvens* YRB2005, *Butyrivibrio fibrisolvens* MD2001, *Kandleria vitulina* WCE2011 and B*utyrivibrio* sp. YAB300

As earlier stated, the LuxS protein was detected in 40.2 % (126 genomes) and 33.0% (45 genomes) of Gram-positive and Gram-negative species respectively, contained within a total of 1711 different genera. Among them, the largest number of LuxS protein genes was detected with the genus *Butyrivibrio* (48 species), followed by *Pseudobutyrivibrio* (15 species), *Prevotella* (14 species) and *Ruminococci* (10 species) (Fig. 2). Furthermore, a phylogenetic tree including the LuxS protein sequences from the 171 putatively positive LuxS species was generated to visualise their similarity. *Vibrio harveyi* and *Streptococcus pneumoniae* LuxS proteins, alongside LuxS proteins identified by Ghali et al. (2016) [9], were used for comparison (Fig. 3). From this phylogenetic tree, it can be observed that LuxS proteins from the same rumen bacterial genera generally clustered together and showed high similarity to each other compared with *Vibrio harveyi* and *Streptococcus pneumoniae*, although multiple clustering of LuxS proteins can occur within the same genera. For instance, LuxS proteins from the *Butyrivibrio* genus formed at least 8 phylogenetically distinct clusters (Fig. 4). Also of note is the fact that LuxS proteins previously encountered in rumen metagenomics and metatranscriptomic datasets [9] are different to those found within the genomes of cultured bacteria used in this study (Fig. 4), indicating that the LuxS proteins have numerous haplotypes in the rumen, perhaps indicative of the importance of this communication system in this ecosystem. It should also be noted that the LuxS protein sequences investigated within Ghali et al.’s study [9] clustered similarily to each other into two separate clades within this study in line with the phylogenetic trees published by the authors. Our study adds significantly more information to this initial publication due to the recent increased availability of rumen bacterial genomes [13].

### Expression of the identified LuxS synthase genes in rumen metatranscriptome datasets

In order to investigate whether the identified LuxS genes within the hungate genomes [16] are actually expressed in the rumen, we investigated their expression within one of the largest rumen metatranscriptome datasets available [16] (105 GB sequencing data). We found expression of 97 (out of 171, 56.7% of the LuxS genes within these datasets (Fig. 4). In order to evaluate expression, we took the average expression across all 20 of the metratranscriptome sequences from the Shi et al. [16] study. On average, *Prevotella* spp. expressed the most LuxS, particularly *Prevotella* sp. TC2-24 (Fig. 4). High expression levels were also seen for LuxS genes originally detected within Ruminococcaceae bacterium P7, Clostridiales bacterium NK3B98, Prevotellaceae bacterium HUN156 and MN60, *Oribacterium* sp. P6A1 and *Butyrivibrio* sp. VCB2001 (Fig. 4). It should, of course, be noted that the expression data will be proportional to the abundance of the bacteria within the metranscriptome datasets and as such they show a snap shot in time, under those particular circumstances, but expression may differ under altering conditions. It also means that whilst we did not see the expression of 74 of the originally identified LuxS genes within the metatranscriptome datasets, it does not mean that they categorically are not expressed and may well be found under different ecological circumstances. The purpose of investigating the expression of the LuxS genes identified within the Hungate collection was to show that most are expressed and therefore are likely used for QS in the rumen.

## Discussion

Bacterial quorum sensing is known to have major implications for bacterial phenotype in pure culture-based studies [22–24]. However, quorum sensing mechanisms in multi-species biofilm communities are poorly understood due to the complexities of these microbiomes. In this study, we used rumen bacterial genomes from the Hungate collection, a large database which is largely representative of many of the rumen microorganisms (501 bacterial and archaeal genomes) [13] and complete rumen bacterial genomes from Genbank to prospect for the abundance and distribution of AHL and AI-2-based QS systems. For ease of analysis and data visualisation, the bacteria genomes were categorised according to taxonomic information as well as their Gram staining phenotypes. From a total of 448 bacteria species recorded in the Hungate collection (at the time data was accessed for analysis) and Genbank genomes, 311 species were Gram-positive bacteria and 136 were Gram-positive representing 69.4% and 30.6% of the bacteria used within this study.

We found only one species (*Citrobacter* sp. NLAE-zl-C269) out of 136 bacterial genomes from Gram-negative bacteria analysed possessed an AHL synthase gene. *Citrobacter* spp. have been known to have the ability to hydrolyse cellulose in the rumen and have been reported in non-rumen-based studies to produce 3-hydroxyl type of AHLs [25, 26]. We did, however, also find evidence that the rumen *Citrobacter* spp. genome also possessed AI-2 QS capacity, a phenomenon which to our knowledge has not been shown before in other *Citrobacter* sp.

Approximately 40.2 % and 33.0 % of LuxS genes were identified from Gram-positive and Gram-negative bacterial species respectively. Therefore, our results also confirm that the LuxS proteins may be utilised by both Gram-positive and negative bacteria as previously suggested [27–29]. Approximately 40% of the studied rumen bacterial genomes contained LuxS genes, with these predominating in Gram-positive bacteria (40.5% from the 311 Gram-positive bacterial genomes and 33.1% from the 136 Gram-negative bacterial genomes). Sequence alignment and visualisation using a phylogenetic tree show that the LuxS genes found in the rumen bacterial genomes were highly similar to each other compared with *Vibrio harveyi* or *Streptococcus pneumoniae* LuxS genes, indicating that LuxS genes in the rumen may be highly conserved within each genus but are also distantly related to non-ruminal LuxS genes. Also, noteworthy was the fact that the QS genes found within this study were different to those found by Ghali et al. (2016) [9], which may be consequences of the large number of genomes prospected in this study. Irrespectively, the presence of numerous LuxS haplotypes suggests that this QS system may be especially important for bacterial communication within the rumen.

Overall, we show evidence that *Butyrivibrio*, *Prevotella*, *Ruminococcus* and *Pseudobutyrivibrio* rumen bacterial genomes, which are concomitantly the most abundant bacterial genera in the rumen have the capacity to use AI-2-based quorum sensing. This data also suggests that quorum sensing is ubiquitous within the rumen in general. This observation is in agreement with those from many recent studies, which show that these bacterial genera may possess AI-2 QS signalling ability [9–11]. Further observations from this study suggest that bacteria with LuxS protein predominate in the rumen, and it has become clear that the AI-2 signal has a great preponderance in the rumen. The expression results for the identified LuxS genes within large published metatranscriptome datasets [16] confirmed that 97 of the 171 identified LuxS genes were expressed in the rumen. This data also showed that rumen *Prevotella* likely play a major role in AI-2-based QS in the rumen. It has also been shown that oral *Prevotella *sp. possesses AI-2 QS capacity [30].

However, many ruminant bacteria are yet to be identified, cultured or genome sequenced, and further studies to identify them and their potential involvement in QS in the rumen are paramount. It may also be important to assess the contribution of other rumen organism types including fungi, archaea, protozoa and viruses to QS in the rumen and their role in plant degradation alongside bacteria given that several non-ruminal studies show that other microorganisms such as fungi, archaea and protozoa also use QS signalling [31–36]. Moreover, there are still a lot of Gram-negative bacteria that remain uncultured with the potential to utilise AHL-based QS in the rumen. Thus, it is important to improve our knowledge of both AI-2 and AHL QS signalling system in the rumen to better understand bacterial interactions and consequences on ruminant production. Further, in vitro analysis to confirm the bioluminescence activity of the LuxS genes detected in these rumen bacteria genera will be required to understand their expression and role in the rumen microbiome.

## Conclusion

Using the major genomic resources available, we show on a greater scale than previously possible that QS is likely to be an important phenomenon in the rumen microbiome. Our data suggest that AI-2-based QS is probably the most abundant and perhaps most important signal used by rumen bacteria. Further research into the implications of rumen-based QS on plant degradation and nutrient availability for the host is now required.

## Supplementary information

**Additional file 1: Table S1.** Bacterial diversity and the presence of Quorum Sensing autoinducer proteins in the rumen. Among the Gram-positive bacteria, there are three species, which show variable Gram stain (described with weakly+ and variable+). In the Gram-negative section, only one species had detectable AHL synthase genes (highlighted with a grey colour).
