# Supplementary material for: Can rumen bacteria communicate to each other?
Source: Microbiome. 2020 Feb 21;8:23. doi: 10.1186/s40168-020-00796-y (PMC7035670; doi:10.1186/s40168-020-00796-y)
Supplement: Supplementary file 1 — Additional file 1: Table S1. Bacterial diversity and the presence of Quorum Sensing autoinducer proteins in the rumen. Among the Gram-positive bacteria, there are three species, which show variable Gram stain (described with weakly+ and variable+). In the Gram-negative section, only one species had detectable AHL synthase genes (highlighted with a grey colour). [file 40168_2020_796_MOESM1_ESM.docx]

Additional file 1: Table S1. Bacterial diversity and the presence of Quorum Sensing autoinducer proteins in the rumen. Among the Gram-positive bacteria, there are three species, which show variable Gram stain (described with weakly+ and variable+). In the Gram-negative section, only one species had detectable AHL synthase genes (highlighted with a grey colour).

|  | **Species** | **Accession Number for AI protein (NCBI or UniProtKB)** | | **Autoinducer protein identified** |  |
| --- | --- | --- | --- | --- | --- |
| **Gram+** | *Acetitomaculum ruminis DSM 5522* | SFB39308.1 | | S-ribosylhomocysteine lyase /quorum-sensing autoinducer 2 (AI-2) synthesis protein LuxS [*Acetitomaculum ruminis* DSM 5522] |  |
|  |  | SFB40160.1 | | regulatory protein, LuxR family [*Acetitomaculum ruminis* DSM 5522] |  |
|  | *Actinomyces denticolens* PA |  | | N/A |  |
|  | *Actinomyces nasicola* KPR-1 |  | | N/A |  |
|  | *Actinomyces ruminicola* DSM 27982 |  | | N/A |  |
|  | *Actinomyces ruminicola* KPR-7B |  | | N/A |  |
|  | *Bacillus cereus* KPR-7A |  | | N/A |  |
|  | *Bacillus licheniformis* VTM3R78 |  | | N/A |  |
|  | *Bacillus* sp. MB2021 | WP_031539940.1 | | N-acyl homoserine lactonase family protein [*Bacillus* sp. MB2021] |  |
|  | *Bifidobacterium adolescentis* DSM 20087 |  | | N/A |  |
|  | *Bifidobacterium bifidum* Calf96 |  | | N/A |  |
|  | *Bifidobacterium boum* DSM 20432 |  | | N/A |  |
|  | *Bifidobacterium breve* RP2 |  | | N/A |  |
|  | *Bifidobacterium longum* AGR2137 | WP_007053686.1 | | S-ribosylhomocysteine lyase [*Bifidobacterium longum*] |  |
|  | *Bifidobacterium merycicum* DSM 6492 | SHE27967.1 | | S-ribosylhomocysteine lyase /quorum-sensing autoinducer 2 (AI-2) synthesis protein LuxS [*Bifidobacterium merycicum* DSM 6492] |  |
|  |  | SHE30051.1 | | two component transcriptional regulator, LuxR family [*Bifidobacterium merycicum* DSM 6492] |  |
|  | *Bifidobacterium pseudolongum* AGR2145 |  | | N/A |  |
|  | *Bifidobacterium pseudolongum globosum* DSM 20092 | |  | N/A |  |
|  | *Bifidobacterium ruminantium* DSM 6489 |  | | N/A |  |
|  | *Bifidobacterium* sp. AGR2158 |  | | N/A |  |
|  | *Bifidobacterium* *thermophilum* DSM 20212 |  | | N/A |  |
|  | *Blautia schinkii* DSM 10518 | WP_044940107.1 | | S-ribosylhomocysteine lyase [*Blautia schinkii*] |  |
|  |  | WP_084171859.1 | | LuxR family transcriptional regulator [*Blautia schinkii*] |  |
|  |  | WP_044938452.1 | | N-acyl homoserine lactonase family protein [*Blautia schinkii*] |  |
|  | *Blautia* sp. SF-50 | SCX85778.1 | | S-ribosylhomocysteine lyase /quorum-sensing autoinducer 2 (AI-2) synthesis protein LuxS [*Blautia* sp. SF-50] |  |
|  |  | WP_092073512.1 | | LuxR family transcriptional regulator [*Blautia* sp. SF-50] |  |
|  | *Blautia wexlerae* AGR2146 | WP_081678955.1 | | cyclic lactone autoinducer peptide [*Blautia wexlerae*] |  |
|  | *Butyrivibrio fibrisolvens* | WP_022758690.1 | | S-ribosylhomocysteine lyase [*Butyrivibrio fibrisolvens*] |  |
|  |  | SES39897.1 | | two component transcriptional regulator, LuxR family [*Butyrivibrio fibrisolvens*] |  |
|  |  | WP_081357172.1 | | yclic lactone autoinducer peptide [*Butyrivibrio fibrisolvens*] |  |
|  | *Butyrivibrio fibrisolvens* AB2020 | WP_022758690.1 | | S-ribosylhomocysteine lyase [*Butyrivibrio fibrisolvens*] |  |
|  |  | WP_022758261.1 | | DNA-binding response regulator [*Butyrivibrio fibrisolvens*] |  |
|  | *Butyrivibrio fibrisolvens* AR40 |  | | N/A |  |
|  | *Butyrivibrio fibrisolvens* D1 |  | | N/A |  |
|  | *Butyrivibrio fibrisolvens* FE2007 | WP_027203956.1 | | S-ribosylhomocysteine lyase [*Butyrivibrio fibrisolvens*] |  |
|  |  | WP_027204460.1 | | DNA-binding response regulator [*Butyrivibrio fibrisolvens*] |  |
|  |  | WP_027204242.1 | | hypothetical protein [*Butyrivibrio fibrisolvens*] |  |
|  | *Butyrivibrio fibrisolvens* MD2001 | WP_027216398.1 | | S-ribosylhomocysteine lyase [*Butyrivibrio fibrisolvens*] |  |
|  |  | WP_027216302.1 | | DNA-binding response regulator [*Butyrivibrio fibrisolvens*] |  |
|  | *Butyrivibrio fibrisolvens* ND3005 | WP_022753040.1 | | S-ribosylhomocysteine lyase [*Butyrivibrio fibrisolvens*] |  |
|  | *Butyrivibrio fibrisolvens* WTE3004 | WP_027207614.1 | | S-ribosylhomocysteine lyase [*Butyrivibrio fibrisolvens*] |  |
|  | *Butyrivibrio fibrisolvens* YRB2005 | WP_027207614.1 | | S-ribosylhomocysteine lyase [*Butyrivibrio fibrisolvens*] |  |
|  | *Butyrivibrio hungatei* NK4A153 | WP_026656280.1 | | MULTISPECIES: S-ribosylhomocysteine lyase [*Butyrivibrio*] |  |
|  |  | WP_026655111.1 | | MULTISPECIES: cyclic lactone autoinducer peptide [*Butyrivibrio*] |  |
|  | *Butyrivibrio hungatei* XBD2006 |  | | N/A |  |
|  | *Butyrivibrio proteoclasticus* | WP_026663048.1 | | S-ribosylhomocysteine lyase [*Butyrivibrio proteoclasticus*] |  |
|  | *Butyrivibrio proteoclasticus* FD2007 | WP_026663048.1 | | S-ribosylhomocysteine lyase [*Butyrivibrio proteoclasticus*] |  |
|  | *Butyrivibrio proteoclasticus* P18 |  | | N/A |  |
|  | *Butyrivibrio proteoclasticus* P6B7 | WP_026651396.1 | | S-ribosylhomocysteine lyase [*Butyrivibrio proteoclasticus*] |  |
|  | *Butyrivibrio* sp. AC2005 | WP_026658771.1 | | S-ribosylhomocysteine lyase [*Butyrivibrio* sp. AC2005] |  |
|  |  | WP_026658685.1 | | LuxR family transcriptional regulator [*Butyrivibrio* sp. AC2005] |  |
|  | *Butyrivibrio* sp. ACTF2 |  | | N/A |  |
|  | *Butyrivibrio* sp. AD3002 | WP_022762564.1 | | S-ribosylhomocysteine lyase [*Butyrivibrio* sp. AD3002] |  |
|  | *Butyrivibrio* sp. AE2005 | WP_026656280.1 | | MULTISPECIES: S-ribosylhomocysteine lyase [*Butyrivibrio*] |  |
|  |  | WP_026655111.1 | | MULTISPECIES: cyclic lactone autoinducer peptide [*Butyrivibrio*] |  |
|  | *Butyrivibrio* sp. AE2015 | WP_026663688.1 | | MULTISPECIES: S-ribosylhomocysteine lyase [*Butyrivibrio*] |  |
|  | *Butyrivibrio* sp. AE2032 | WP_034445133.1 | | S-ribosylhomocysteine lyase [*Butyrivibrio* sp. AE2032] |  |
|  | *Butyrivibrio* sp. AE3003 | WP_026656280.1 | | MULTISPECIES: S-ribosylhomocysteine lyase [*Butyrivibrio*] |  |
|  |  | WP_026655111.1 | | MULTISPECIES: cyclic lactone autoinducer peptide [*Butyrivibrio*] |  |
|  | *Butyrivibrio* sp. AE3004 | WP_035777305.1 | | S-ribosylhomocysteine lyase [*Butyrivibrio* sp. AE3004] |  |
|  | *Butyrivibrio* sp. AE3006 | WP_026670344.1 | | S-ribosylhomocysteine lyase [*Butyrivibrio* sp. AE3006] |  |
|  |  | WP_026670784.1 | | LuxR family transcriptional regulator [*Butyrivibrio* sp. AE3006] |  |
|  | *Butyrivibrio* sp. AE3009 | WP_022777361.1 | | S-ribosylhomocysteine lyase [*Butyrivibrio* sp. AE3009] |  |
|  | *Butyrivibrio* sp. FB3011 |  | | N/A |  |
|  | *Butyrivibrio* sp. FC2001 | WP_026492333.1 | | MULTISPECIES: S-ribosylhomocysteine lyase [*Butyrivibrio*] |  |
|  |  | WP_081673411.1 | | LuxR family transcriptional regulator [*Butyrivibrio* sp. FC2001] |  |
|  | *Butyrivibrio* sp. FCS006 | WP_035770679.1 | | S-ribosylhomocysteine lyase [*Butyrivibrio* sp. FCS006] |  |
|  |  | WP_081664776.1 | | hypothetical protein [*Butyrivibrio* sp. FCS006] |  |
|  | *Butyrivibrio* sp. FCS014 | WP_024866099.1 | | S-ribosylhomocysteine lyase [*Butyrivibrio* sp. FCS014] |  |
|  | *Butyrivibrio* sp. INlla14 | SCX91844.1 | | S-ribosylhomocysteine lyase /quorum-sensing autoinducer 2 (AI-2) synthesis protein LuxS [*Butyrivibrio* sp. INlla14] |  |
|  | *Butyrivibrio* sp. INlla16 | SDB63310.1 | | S-ribosylhomocysteine lyase /quorum-sensing autoinducer 2 (AI-2) synthesis protein LuxS [*Butyrivibrio* sp. INlla16] |  |
|  |  | SDB66303.1 | | Motility quorum-sensing regulator, toxin of MqsA [*Butyrivibrio* sp. INlla16] |  |
|  | *Butyrivibrio* sp. INlla18 | SDA70346.1 | | S-ribosylhomocysteine lyase /quorum-sensing autoinducer 2 (AI-2) synthesis protein LuxS [*Butyrivibrio* sp. INlla18] |  |
|  |  | SDA55440.1 | | regulatory protein, LuxR family [*Butyrivibrio* sp. INlla18] |  |
|  | *Butyrivibrio* sp. INlla21 | SFU62968.1 | | S-ribosylhomocysteine lyase /quorum-sensing autoinducer 2 (AI-2) synthesis protein LuxS [*Butyrivibrio* sp. INlla21] |  |
|  | *Butyrivibrio* sp. LB2008 | WP_026513449.1 | | S-ribosylhomocysteine lyase [*Butyrivibrio* sp. LB2008] |  |
|  |  | WP_026512765.1 | | cyclic lactone autoinducer peptide [*Butyrivibrio* sp. LB2008] |  |
|  | *Butyrivibrio* sp. LC3010 | WP_026510091.1 | | MULTISPECIES: S-ribosylhomocysteine lyase [*Butyrivibrio*] |  |
|  | *Butyrivibrio* sp. M55 | SFU54075.1 | | S-ribosylhomocysteine lyase /quorum-sensing autoinducer 2 (AI-2) synthesis protein LuxS [*Butyrivibrio* sp. M55] |  |
|  |  | WP_092338062.1 | | cyclic lactone autoinducer peptide [*Butyrivibrio* sp. M55] |  |
|  | *Butyrivibrio* sp. MB2005 | WP_026524524.1 | | S-ribosylhomocysteine lyase [*Butyrivibrio* sp. MB2005] |  |
|  | *Butyrivibrio* sp. MC2013 |  | | N/A |  |
|  | *Butyrivibrio* sp. MC2021 | WP_026517760.1 | | S-ribosylhomocysteine lyase [*Butyrivibrio* sp. MC2021] |  |
|  |  | WP_026517696.1 | | LuxR family transcriptional regulator [*Butyrivibrio* sp. MC2021] |  |
|  |  | WP_081795621.1 | | cyclic lactone autoinducer peptide [*Butyrivibrio* sp. MC2021] |  |
|  | *Butyrivibrio* sp. NC2002 | WP_035765513.1 | | S-ribosylhomocysteine lyase [*Butyrivibrio* sp. NC2002] |  |
|  | *Butyrivibrio* sp. NC2007 | WP_022769324.1 | | S-ribosylhomocysteine lyase [*Butyrivibrio* sp. NC2007] |  |
|  | *Butyrivibrio* sp. NC3005 | WP_026505527.1 | | S-ribosylhomocysteine lyase [*Butyrivibrio* sp. NC3005] |  |
|  | *Butyrivibrio* sp. OB235 | SEM43779.1 | | S-ribosylhomocysteine lyase /quorum-sensing autoinducer 2 (AI-2) synthesis protein LuxS [*Butyrivibrio* sp. ob235] |  |
|  |  | SEL05264.1 | | two component transcriptional regulator, LuxR family [*Butyrivibrio* sp. ob235] |  |
|  | *Butyrivibrio* sp. OB251 |  | | N/A |  |
|  | *Butyrivibrio* sp. Su6 | SEG36042.1 | | S-ribosylhomocysteine lyase /quorum-sensing autoinducer 2 (AI-2) synthesis protein LuxS [*Butyrivibrio* sp. Su6] |  |
|  |  | SEF45072.1 | | LuxR family transcriptional regulator, maltose regulon positive regulatory protein [*Butyrivibrio* sp. Su6] |  |
|  | *Butyrivibrio* sp. TB | SEP65807.1 | | S-ribosylhomocysteine lyase /quorum-sensing autoinducer 2 (AI-2) synthesis protein LuxS [*Butyrivibrio* sp. TB] |  |
|  |  | SEQ17893.1 | | cyclic lactone autoinducer peptide [*Butyrivibrio* sp. TB] |  |
|  | *Butyrivibrio* sp. VCB2001 | WP_026521437.1 | | S-ribosylhomocysteine lyase [*Butyrivibrio* sp. VCB2001] |  |
|  | *Butyrivibrio* sp. VCB2006 | WP_029231439.1 | | S-ribosylhomocysteine lyase [*Butyrivibrio* sp. VCB2006] |  |
|  | *Butyrivibrio* sp. VCD2006 | WP_026526228.1 | | S-ribosylhomocysteine lyase [*Butyrivibrio* sp. VCD2006] |  |
|  | *Butyrivibrio* sp. WCD2001 | WP_026498888.1 | | MULTISPECIES: S-ribosylhomocysteine lyase [*Butyrivibrio*] |  |
|  | *Butyrivibrio* sp. WCD3002 | WP_035794944.1 | | S-ribosylhomocysteine lyase [*Butyrivibrio* sp. WCD3002] |  |
|  | *Butyrivibrio* sp. WCE2006 | WP_026510091.1 | | MULTISPECIES: S-ribosylhomocysteine lyase [*Butyrivibrio*] |  |
|  | *Butyrivibrio* sp. XBB1001 | WP_026489236.1 | | S-ribosylhomocysteine lyase [*Butyrivibrio* sp. XBB1001] |  |
|  |  | WP_026488770.1 | | LuxR family transcriptional regulator [*Butyrivibrio* sp. XBB1001] |  |
|  | *Butyrivibrio* sp. XPD2002 | WP_026492333.1 | | MULTISPECIES: S-ribosylhomocysteine lyase [*Butyrivibrio*] |  |
|  | *Butyrivibrio* sp. XPD2006 | WP_026493991.1 | | S-ribosylhomocysteine lyase [*Butyrivibrio* sp. XPD2006] |  |
|  |  | WP_022763694.1 | | DNA-binding response regulator [*Butyrivibrio* sp. XPD2006] |  |
|  | *Butyrivibrio* sp. YAB3001 | SFC84424.1 | | S-ribosylhomocysteine lyase /quorum-sensing autoinducer 2 (AI-2) synthesis protein LuxS [*Butyrivibrio* sp. YAB3001] |  |
|  |  | SFC30639.1 | | regulatory protein, LuxR family [*Butyrivibrio* sp. YAB3001] |  |
|  |  | SFC12536.1 | | N-acyl homoserine lactone hydrolase [*Butyrivibrio* sp. YAB3001] |  |
|  |  | WP_141107693.1 | | cyclic lactone autoinducer peptide [*Butyrivibrio* sp. YAB3001] |  |
|  | *Cellulomonas* sp. KH9 | SFK30379.1 | | S-ribosylhomocysteine lyase /quorum-sensing autoinducer 2 (AI-2) synthesis protein LuxS [*Cellulomonas* sp. KH9] |  |
|  |  | SFJ82371.1 | | LuxR family transcriptional regulator [*Cellulomonas* sp. KH9] |  |
|  | *Clostridiales bacterium* NK3B98 | WP_022786818.1 | | S-ribosylhomocysteine lyase [*Clostridiales bacterium* NK3B98] |  |
|  |  | WP_022787663.1 | | LuxR family transcriptional regulator [*Clostridiales bacterium* NK3B98] |  |
|  | *Clostridiales bacterium* R-7 |  | | N/A |  |
|  | *Clostridiales bacterium* WTE2008 |  | | N/A |  |
|  | *Clostridium aerotolerans* DSM 5434 | WP_026891040.1 | | S-ribosylhomocysteine lyase [[*Clostridium*] *aerotolerans*] |  |
|  |  | WP_084326176.1 | | cyclic lactone autoinducer peptide [[*Clostridium*] *aerotolerans*] |  |
|  |  | WP_051629853.1 | | LuxR family transcriptional regulator [[*Clostridium*] *aerotolerans*] |  |
|  | *Clostridium* *algidicarnis* B3 |  | | N/A |  |
|  | *Clostridium* *aminophilum* F |  | | N/A |  |
|  | *Clostridium* *beijerinckii* HUN142 | WP_026889258.1 | | MULTISPECIES: S-ribosylhomocysteine lyase [*Clostridium*] |  |
|  |  | WP_026889071.1 | | LuxR family transcriptional regulator [*Clostridium* *beijerinckii*] |  |
|  |  | WP_080697131.1 | | cyclic lactone autoinducer peptide [*Clostridium* *beijerinckii*] |  |
|  | *Clostridium* *butyricum* AGR2140 | WP_002581777.1 | | MULTISPECIES: S-ribosylhomocysteine lyase [*Clostridium*] |  |
|  |  | WP_003428320.1 | | LuxR family transcriptional regulator [*Clostridium* *butyricum*] |  |
|  |  | WP_080658001.1 | | cyclic lactone autoinducer peptide [*Clostridium* *butyricum*] |  |
|  |  | WP_027635219.1 | | autoinducer-2 kinase [*Clostridium* *butyricum*] |  |
|  | *Clostridium* *cadaveris* AGR2141 | WP_035770819.1 | | cyclic lactone autoinducer peptide [*Clostridium* *cadaveris*] |  |
|  |  | WP_027638993.1 | | DNA-binding response regulator [*Clostridium* *cadaveris*] |  |
|  | *Clostridium* *cadaveris* NLAE-zl-G419 |  | | N/A |  |
|  | *Clostridium* *clostidiforme* ATCC 25537 |  | | N/A |  |
|  | *Clostridium* *clostridioforme* AGR2157 | WP_027641561.1 | | S-ribosylhomocysteine lyase [[*Clostridium*] *clostridioforme*] |  |
|  |  | WP_080664557.1 | | cyclic lactone autoinducer peptide [[*Clostridium*] *clostridioforme*] |  |
|  |  | WP_027640567.1 | | MULTISPECIES: autoinducer-2 kinase [*Clostridiales*] |  |
|  | *Clostridium* *cochlearium* NLAE-zl-C224 |  | | N/A |  |
|  | *Clostridium* *glycolicum* CA6 |  | | N/A |  |
|  | *Clostridium* *glycolicum* KPPR-9 |  | | N/A |  |
|  | *Clostridium innocuum* NLAE-zl-C381 |  | | N/A |  |
|  | *Clostridium* *innocuum* NLAE-zl-G197 |  | | N/A |  |
|  | *Clostridium* *lundense* DSM 17049 | WP_084284659.1 | | LuxR family transcriptional regulator [*Clostridium* *lundense*] |  |
|  |  | WP_084284614.1 | | cyclic lactone autoinducer peptide [*Clostridium* *lundense*] |  |
|  | *Clostridium mangenotii* LM2 | WP_027703203.1 | | S-ribosylhomocysteine lyase [*Clostridioides mangenotii*] |  |
|  |  | WP_081800383.1 | | LuxR family transcriptional regulator [*Clostridioides mangenotii*] |  |
|  |  | WP_081800441.1 | | cyclic lactone autoinducer peptide [*Clostridioides mangenotii*] |  |
|  | *Clostridium* *paraputrificum* AGR2156 | WP_034866511.1 | | MULTISPECIES: cyclic lactone autoinducer peptide [*Clostridium*] |  |
|  | *Clostridium* *polysaccharolyticum* DSM1801 |  | | N/A |  |
|  | *Clostridium* sp. DSM8431 | SFU67962.1 | | S-Ribosylhomocysteinase (LuxS) [*Clostridium* sp. DSM 8431] |  |
|  |  | SFU35495.1 | | two component transcriptional regulator, LuxR family [*Clostridium* sp. DSM 8431] |  |
|  |  | WP_090012430.1 | | cyclic lactone autoinducer peptide [*Clostridium* sp. DSM 8431] |  |
|  | *Coriobacteriaceae bacterium* KH1P3 | SJZ37909.1 | | S-ribosylhomocysteine lyase /quorum-sensing autoinducer 2 (AI-2) synthesis protein LuxS [*Olsenella* sp. KH1P3] |  |
|  |  | SJZ65664.1 | | regulatory protein, LuxR family [*Olsenella* sp. KH1P3] |  |
|  |  | SJZ74554.1 | | autoinducer 2 (AI-2) kinase [*Olsenella* sp. KH1P3] |  |
|  | *Corynebacterium vitaeruminis* Ga6A13 | WP_081839972.1 | | LuxR family transcriptional regulator [*Corynebacterium vitaeruminis*] |  |
|  | *Denitrobacterium Detoxificans* DSM 21843 |  | | N/A |  |
|  | *Desulfotomaculum ruminis* | AEG62029.1 | | regulatory protein LuxR [*Desulfotomaculum ruminis* DSM 2154] |  |
|  |  | WP_013843173.1 | | cyclic lactone autoinducer peptide [*Desulfotomaculum ruminis*] |  |
|  |  | AEG61427.1 | | hypothetical protein Desru_3221 [*Desulfotomaculum ruminis* DSM 2154] |  |
|  | *Dorea longicatena* AGR2136 | WP_028085972.1 | | S-ribosylhomocysteine lyase [*Dorea longicatena*] |  |
|  |  | WP_028086355.1 | | N-acyl homoserine lactonase family protein [*Dorea longicatena*] |  |
|  | *Dorea* sp. AGR2135 | WP_022279089.1 | | MULTISPECIES: S-ribosylhomocysteine lyase [*Dorea*] |  |
|  |  | WP_081472126.1 | | MULTISPECIES: cyclic lactone autoinducer peptide [*Dorea*] |  |
|  | *Enterococcus casseliflavus* NLAE-zl-C414 |  | | N/A |  |
|  | *Enterococcus casseliflavus* NLAE-zl-G268 |  | | N/A |  |
|  | *Enterococcus faecalis* 68A |  | | N/A |  |
|  | *Enterococcus faecalis* VTM1R91 |  | | N/A |  |
|  | *Enterococcus gallinarum* SKF1 |  | | N/A |  |
|  | *Enterococcus mundtii* C2 |  | | N/A |  |
|  | *Enterococcus* sp. KPPR-6 | WP_090402604.1 | | LuxR family transcriptional regulator [*Enterococcus* sp. kppr-6] |  |
|  |  | WP_090409084.1 | | cyclic lactone autoinducer peptide [*Enterococcus* sp. kppr-6] |  |
|  |  | SET50089.1 | | autoinducer 2 (AI-2) kinase [*Enterococcus* sp. kppr-6] |  |
|  | *Erysipelotrichaceae bacterium* NK3D112 | WP_034926960.1 | | S-ribosylhomocysteine lyase [*Erysipelotrichaceae bacterium* NK3D112] |  |
|  | *Eubacterium* *callanderi* NLAE-zl-G225 |  | | N/A |  |
|  | *Eubacterium* *cellulosolvens* LD2006 | WP_027870715.1 | | S-ribosylhomocysteine lyase [[*Eubacterium*] *cellulosolvens*] |  |
|  |  | WP_081794715.1 | | LuxR family transcriptional regulator [[*Eubacterium*] *cellulosolvens*] |  |
|  |  | WP_027870444.1 | | autoinducer 2 ABC transporter substrate-binding protein [[*Eubacterium*] *cellulosolvens*] |  |
|  | *Eubacterium* *eligens* | CUQ83259.1 | | S-ribosylhomocysteine lyase [[*Eubacterium*] *eligens*] |  |
|  |  | ACR72038.1 | | LuxR family transcriptional regulator, maltose regulon positive regulatory protein [[*Eubacterium*] *eligens* ATCC 27750] |  |
|  |  | CUQ79865.1 | | transcriptional regulator MalT [[*Eubacterium*] *eligens*] |  |
|  |  | ACR73040.1 | | two-component system, NarL family, response regulator DegU [[*Eubacterium*] *eligens* ATCC 27750] |  |
|  |  | CUQ90545.1 | | Response regulator protein vraR [[*Eubacterium*] *eligens*] |  |
|  |  | WP_082414136.1 | | cyclic lactone autoinducer peptide [[*Eubacterium*] *eligens*] |  |
|  |  | ACR71661.1 | | Hypothetical protein EUBELI_00648 [[*Eubacterium*] *eligens* ATCC 27750] |  |
|  | *Eubacterium* *limosum* P2 |  | | N/A |  |
|  | *Eubacterium* *oxidoreducens* DSM 3217 |  | | N/A |  |
|  | *Eubacterium* *pyruvativorans* KHGC13 |  | | N/A |  |
|  | *Eubacterium* *pyruvativorans* KHPC4 |  | | N/A |  |
|  | *Eubacterium* *pyruvativorans* i6 |  | | N/A |  |
|  | *Eubacterium rectalis* | CUN26990.1 | | S-ribosylhomocysteine lyase [[*Eubacterium*] *rectale*] |  |
|  |  | RHC40766.1 | | LuxR family transcriptional regulator [[*Eubacterium*] *rectale*] |  |
|  |  | CUO30060.1 | | Response regulator protein vraR [[*Eubacterium*] *rectale*] |  |
|  |  | WP_055273834.1 | | hypothetical protein [[*Eubacterium*] *rectale*] |  |
|  |  | ACR73916.1 | | probable carbon storage regulator [[*Eubacterium rectale*] ATCC 33656] |  |
|  |  | WP_015516101.1 | | cyclic lactone autoinducer peptide [[*Eubacterium*] *rectale*] |  |
|  | *Eubacterium* *ruminantium* 2388 |  | | N/A |  |
|  | *Eubacterium* *ruminantium* FB3002 |  | | N/A |  |
|  | *Eubacterium* *ruminantium* GA 195 |  | | N/A |  |
|  | *Eubacterium* *ruminatium* HUN269 |  | | N/A |  |
|  | *Eubacterium* *saphenum* |  | | N/A |  |
|  | *Eubacterium* sp. AB3007 | WP_027869703.1 | | LuxR family transcriptional regulator [*Eubacterium* sp. AB3007] |  |
|  | *Holdemanella biformis* | EEC89883.1 | | S-ribosylhomocysteinase LuxS [*Holdemanella biformis* DSM 3989] |  |
|  |  | RGW74953.1 | | LuxR family transcriptional regulator [*Holdemanella biformis*] |  |
|  |  | RGW72764.1 | | cyclic lactone autoinducer peptide [*Holdemanella biformis*] |  |
|  |  | EEC90479.1 | | hypothetical protein EUBIFOR_00946 [*Holdemanella biformis* DSM 3989] |  |
|  | *Kandleria vitulina* DSM 20405 | WP_029069890.1 | | S-ribosylhomocysteine lyase [*Kandleria vitulina*] |  |
|  |  | KRN47404.1 | | two-component system response regulator [*Kandleria vitulina* DSM 20405] |  |
|  | *Kandleria vitulina* KH4T7 |  | | N/A |  |
|  | *Kandleria vitulina* MC3001 | WP_029069890.1 | | S-ribosylhomocysteine lyase [*Kandleria vitulina*] |  |
|  | *Kandleria vitulina* S3b |  | | N/A |  |
|  | *Kandleria vitulina* WCC7 |  | | N/A |  |
|  | *Kandleria vitulina* WCE2011 | WP_029069890.1 | | S-ribosylhomocysteine lyase [*Kandleria vitulina*] |  |
| **weakly +** | *Lachnobacterium bovis* AE2004 |  | | N/A |  |
| **weakly +** | *Lachnobacterium bovis* C6A12 | WP_027421877.1 | | S-ribosylhomocysteine lyase [*Lachnobacterium bovis*] |  |
|  |  | WP_022750197.1 | | cyclic lactone autoinducer peptide [*Lachnobacterium bovis*] |  |
| **weakly +** | *Lachnobacterium bovis* DSM 14045 | SDY67808.1 | | S-ribosylhomocysteine lyase /quorum-sensing autoinducer 2 (AI-2) synthesis protein LuxS [*Lachnobacterium bovis* DSM 14045] |  |
|  |  | SDY79097.1 | | cyclic lactone autoinducer peptide [*Lachnobacterium bovis* DSM 14045] |  |
| **weakly +** | *Lachnobacterium bovis* NK4B19 | WP_022749352.1 | | S-ribosylhomocysteine lyase [*Lachnobacterium bovis*] |  |
|  |  | WP_022750197.1 | | cyclic lactone autoinducer peptide [*Lachnobacterium bovis*] |  |
| **weakly +** | *Lachnobacterium bovis* S1b |  | | N/A |  |
|  | *Lachnoclostridium aminophilum* KH1P1 |  | | N/A |  |
|  | *Lachnoclostridium citroniae* NLAE-zl-G70 |  | | N/A |  |
|  | *Lachnoclostridium* *clostridioforme* NLAE-zl-C196 |  | | N/A |  |
|  | *Lachnoclostridium* *clostridioforme* NLAE-zl-G208 |  | | N/A |  |
|  | *Lachnoclostridium* *contortum* NLAE-zl-C134 |  | | N/A |  |
|  | *Lachnoclostridium lavalense* NLAE-zl-G277 |  | | N/A |  |
|  | *Lachnoclostridium* *oroticum* NLAE-zl-C242 |  | | N/A |  |
|  | *Lachnospira multipara* ATCC 19207 | WP_027431934.1 | | MULTISPECIES: S-ribosylhomocysteine lyase [*Lachnospira*] |  |
|  |  | WP_027430741.1 | | LuxR family transcriptional regulator [*Lachnospira multipara*] |  |
|  | *Lachnospira multipara* D15d |  | | N/A |  |
|  | *Lachnospira multipara* LB2003 | WP_027437165.1 | | S-ribosylhomocysteine lyase [*Lachnospira multipara*] |  |
|  |  | WP_031555876.1 | | LuxR family transcriptional regulator [*Lachnospira multipara*] |  |
|  | *Lachnospira multipara* MC2003 | WP_027437165.1 | | S-ribosylhomocysteine lyase [*Lachnospira multipara*] |  |
|  |  | WP_027437512.1 | | LuxR family transcriptional regulator [*Lachnospira multipara*] |  |
|  | *Lachnospira* *pectinoschiza* M46 |  | | N/A |  |
|  | *Lachnospira* *pectinoschiza* M83 |  | | N/A |  |
|  | *Lachnospiraceae* *bacterium* A10 | SEI89630.1 | | S-ribosylhomocysteine lyase /quorum-sensing autoinducer 2 (AI-2) synthesis protein LuxS [*Lachnospiraceae* *bacterium* A10] |  |
|  | *Lachnospiraceae* *bacterium* AB2028 |  | | N/A |  |
|  | *Lachnospiraceae* *bacterium* AC2012 | WP_027432681.1 | | MULTISPECIES: S-ribosylhomocysteine lyase [unclassified *Lachnospiraceae*] |  |
|  | *Lachnospiraceae* *bacterium* AC2014 | WP_031546102.1 | | S-ribosylhomocysteine lyase [*Lachnospiraceae* *bacterium* AC2014] |  |
|  | *Lachnospiraceae* *bacterium* AC2028 | WP_031581960.1 | | S-ribosylhomocysteine lyase [*Lachnospiraceae* *bacterium* AC2028] |  |
|  | *Lachnospiraceae* *bacterium* AC2029 | WP_034232582.1 | | S-ribosylhomocysteine lyase [*Lachnospiraceae* *bacterium* AC2029] |  |
|  | *Lachnospiraceae* *bacterium* AC2031 |  | | N/A |  |
|  | *Lachnospiraceae* *bacterium* AC3007 | WP_027424153.1 | | LuxR family transcriptional regulator [*Lachnospiraceae* *bacterium* AC3007] |  |
|  |  | WP_027424445.1 | | autoinducer 2 ABC transporter substrate-binding protein [*Lachnospiraceae* *bacterium* AC3007] |  |
|  | *Lachnospiraceae* *bacterium* AD3010 | WP_027427956.1 | | autoinducer 2 ABC transporter substrate-binding protein [*Lachnospiraceae* *bacterium* AD3010] |  |
|  | *Lachnospiraceae* *bacterium* C6A11 | WP_035628293.1 | | S-ribosylhomocysteine lyase [*Lachnospiraceae* *bacterium* C6A11] |  |
|  | *Lachnospiraceae* *bacterium* C7 | SFG15138.1 | | S-ribosylhomocysteine lyase /quorum-sensing autoinducer 2 (AI-2) synthesis protein LuxS [*Lachnospiraceae* *bacterium* C7] |  |
|  |  | WP_090077640.1 | | hypothetical protein [*Lachnospiraceae* *bacterium* C7] |  |
|  |  | WP_090076916.1 | | cyclic lactone autoinducer peptide [*Lachnospiraceae* *bacterium* C7] |  |
|  | *Lachnospiraceae* *bacterium* C10 | SCW78214.1 | | S-ribosylhomocysteine lyase /quorum-sensing autoinducer 2 (AI-2) synthesis protein LuxS [*Lachnospiraceae* *bacterium* C10] |  |
|  |  | WP_090022154.1 | | hypothetical protein [*Lachnospiraceae* *bacterium* C10] |  |
|  | *Lachnospiraceae* *bacterium* FD2005 | WP_027432681.1 | | MULTISPECIES: S-ribosylhomocysteine lyase [unclassified *Lachnospiraceae*] |  |
|  | *Lachnospiraceae* *bacterium* FE2018 | WP_035645224.1 | | S-ribosylhomocysteine lyase [*Lachnospiraceae* *bacterium* FE2018] |  |
|  |  | WP_035640594.1 | | LuxR family transcriptional regulator [*Lachnospiraceae* *bacterium* FE2018] |  |
|  |  | WP_035644778.1 | | N-acyl homoserine lactonase family protein [*Lachnospiraceae* *bacterium* FE2018] |  |
|  | *Lachnospiraceae* *bacterium* G11 | WP_089868808.1 | | LuxR family transcriptional regulator [*Lachnospiraceae* *bacterium* G11] |  |
|  | *Lachnospiraceae* *bacterium* G41 |  | | N/A |  |
|  | *Lachnospiraceae* *bacterium* HUN248 |  | | N/A |  |
|  | *Lachnospiraceae* *bacterium* KH1P17 |  | | N/A |  |
|  | *Lachnospiraceae* *bacterium* KH1T2 | SFK86129.1 | | two component transcriptional regulator, LuxR family [*Lachnospiraceae* *bacterium* KH1T2] |  |
|  | *Lachnospiraceae* *bacterium* KHCPX20 | SDW65653.1 | | S-ribosylhomocysteine lyase /quorum-sensing autoinducer 2 (AI-2) synthesis protein LuxS [*Lachnospiraceae* *bacterium* KHCPX20] |  |
|  |  | WP_089905340.1 | | hypothetical protein [*Lachnospiraceae* *bacterium* KHCPX20] |  |
|  | *Lachnospiraceae* *bacterium* LC2019 |  | | N/A |  |
|  | *Lachnospiraceae* *bacterium* MA2020 | WP_044917685.1 | | hypothetical protein [*Lachnospiraceae* *bacterium* MA2020] |  |
|  | *Lachnospiraceae bacterium* MC2017 | WP_044910649.1 | | LuxR family transcriptional regulator [*Lachnospiraceae* *bacterium* MC2017] |  |
|  |  | WP_044906519.1 | | autoinducer 2 ABC transporter substrate-binding protein [*Lachnospiraceae* *bacterium* MC2017] |  |
|  | *Lachnospiraceae* *bacterium* MD2004 | WP_027432681.1 | | MULTISPECIES: S-ribosylhomocysteine lyase [unclassified *Lachnospiraceae*] |  |
|  | *Lachnospiraceae* *bacterium* NC2004 | WP_027425349.1 | | S-ribosylhomocysteine lyase [*Lachnospiraceae* *bacterium* NC2004] |  |
|  |  | WP_081779300.1 | | cyclic lactone autoinducer peptide [*Lachnospiraceae* *bacterium* NC2004] |  |
|  | *Lachnospiraceae* *bacterium* NC2008 |  | | N/A |  |
|  | *Lachnospiraceae* *bacterium* ND2006 |  | | N/A |  |
|  | *Lachnospiraceae* *bacterium* ND3006 |  | | N/A |  |
|  | *Lachnospiraceae* *bacterium* NE2001 | SEP90982.1 | | S-ribosylhomocysteine lyase /quorum-sensing autoinducer 2 (AI-2) synthesis protein LuxS [*Lachnospiraceae* *bacterium* NE2001] |  |
|  |  | SEP54837.1 | | two component transcriptional regulator, LuxR family [*Lachnospiraceae* *bacterium* NE2001] |  |
|  | *Lachnospiraceae* *bacterium* NK3A20 | SEA17241.1 | | S-ribosylhomocysteine lyase /quorum-sensing autoinducer 2 (AI-2) synthesis protein LuxS [*Lachnospiraceae* *bacterium* NK3A20] |  |
|  | *Lachnospiraceae* *bacterium* NK4A136 | WP_084683822.1 | | LuxR family transcriptional regulator [*Lachnospiraceae* *bacterium* NK4A136] |  |
|  |  | WP_022782056.1 | | hypothetical protein [*Lachnospiraceae* *bacterium* NK4A136] |  |
|  | *Lachnospiraceae* *bacterium* NK4A144 | WP_027115009.1 | | LuxR family transcriptional regulator [*Lachnospiraceae* *bacterium* NK4A144] |  |
|  |  | WP_027112829.1 | | autoinducer 2 ABC transporter substrate-binding protein [*Lachnospiraceae* *bacterium* NK4A144] |  |
|  |  | WP_027112114.1 | | hypothetical protein [*Lachnospiraceae* *bacterium* NK4A144] |  |
|  | *Lachnospiraceae* *bacterium* NK4A179 | WP_022784721.1 | | hypothetical protein [*Lachnospiraceae* *bacterium* NK4A179] |  |
|  | *Lachnospiraceae* *bacterium* NLAE-zl-G231 | SFH60847.1 | | two component transcriptional regulator, LuxR family [*Lachnospiraceae* *bacterium* NLAE-zl-G231] |  |
|  |  | WP_089981878.1 | | autoinducer 2 ABC transporter substrate-binding protein [*Lachnospiraceae* *bacterium* NLAE-zl-G231] |  |
|  |  | WP_089981813.1 | | cyclic lactone autoinducer peptide [unclassified *Lachnospiraceae*] |  |
|  | *Lachnospiraceae* *bacterium* P6A3 | WP_031585120.1 | | S-ribosylhomocysteine lyase [*Lachnospiraceae* *bacterium* P6A3] |  |
|  | *Lachnospiraceae* *bacterium* P6B14 | WP_027115200.1 | | S-ribosylhomocysteine lyase [*Lachnospiraceae* *bacterium* P6B14] |  |
|  | *Lachnospiraceae* *bacterium* RM5 | SEQ34636.1 | | S-ribosylhomocysteine lyase /quorum-sensing autoinducer 2 (AI-2) synthesis protein LuxS [*Lachnospiraceae* *bacterium* RM5] |  |
|  | *Lachnospiraceae* *bacterium* V9D3004 | WP_027105300.1 | | S-ribosylhomocysteine lyase [*Lachnospiraceae* *bacterium* V9D3004] |  |
|  |  | WP_027104513.1 | | LuxR family transcriptional regulator [*Lachnospiraceae* *bacterium* V9D3004] |  |
|  |  | WP_081783396.1 | | cyclic lactone autoinducer peptide [*Lachnospiraceae* *bacterium* V9D3004] |  |
|  | *Lachnospiraceae* *bacterium* XBB1006 |  | | N/A |  |
|  | *Lachnospiraceae* *bacterium* XBB2008 | WP_089855945.1 | | LuxR family transcriptional regulator [*Lachnospiraceae* *bacterium* XBB2008] |  |
|  | *Lachnospiraceae* *bacterium* XBD2001 | SFT33178.1 | | S-ribosylhomocysteine lyase /quorum-sensing autoinducer 2 (AI-2) synthesis protein LuxS [*Lachnospiraceae* *bacterium* XBD2001] |  |
|  |  | WP_090145251.1 | | hypothetical protein [*Lachnospiraceae* *bacterium* XBD2001] |  |
|  | *Lachnospiraceae* *bacterium* XPB1003 |  | | N/A |  |
|  | *Lachnospiraceae* *bacterium* YSB2008 | WP_027118902.1 | | S-ribosylhomocysteine lyase [*Lachnospiraceae* *bacterium* YSB2008] |  |
|  |  | WP_081817142.1 | | LuxR family transcriptional regulator [*Lachnospiraceae* *bacterium* YSB2008] |  |
|  | *Lachnospiraceae* *bacterium* YSD2013 | WP_090036577.1 | | LuxR family transcriptional regulator [*Lachnospiraceae* *bacterium* YSD2013] |  |
|  | *Lactobacillus* *brevis* AG48 |  | | N/A |  |
|  | *Lactobacillus mucosae* AGR63 |  | | N/A |  |
|  | *Lactobacillus mucosae* KHPC15 |  | | N/A |  |
|  | *Lactobacillus* *mucosae* KHPX11 |  | | N/A |  |
|  | *Lactobacillus mucosae* WCC8 |  | | N/A |  |
|  | *Lactobacillus plantarum* AG30 |  | | N/A |  |
|  | *Lactobacillus ruminis* | SEM71308.1 | | S-ribosylhomocysteine lyase /quorum-sensing autoinducer 2 (AI-2) synthesis protein LuxS [*Lactobacillus ruminis*] |  |
|  |  | SEM50488.1 | | two component transcriptional regulator, LuxR family [*Lactobacillus ruminis*] |  |
|  | *Lactobacillus ruminis* DSM 20403 | SFG33971.1 | | S-ribosylhomocysteine lyase /quorum-sensing autoinducer 2 (AI-2) synthesis protein LuxS [*Lactobacillus* *ruminis* DSM 20403 = NBRC 102161] |  |
|  | *Lactobacillus ruminis* WC1T17 |  | | N/A |  |
|  | *Lactococcus garvieae* M79 |  | | N/A |  |
|  | *Lactococcus lactis* subsp. *lactis* 511 |  | | N/A |  |
|  | *Micrococcineae* *bacterium* KH10 |  | | N/A |  |
| **variable +** | *Micrococcus luteus* VTM4R57 |  | | N/A |  |
|  | *Olsenella* sp. KH2P3 | SFX01370.1 | | S-ribosylhomocysteine lyase /quorum-sensing autoinducer 2 (AI-2) synthesis protein LuxS [*Olsenella* sp. kh2p3] |  |
|  |  | WP_083432309.1 | | LuxR family transcriptional regulator [*Olsenella* sp. kh2p3] |  |
|  | *Olsenella* sp. KH3B4 | SES84064.1 | | S-ribosylhomocysteine lyase /quorum-sensing autoinducer 2 (AI-2) synthesis protein LuxS [*Olsenella* sp. KH3B4] |  |
|  |  | WP_091005835.1 | | LuxR family transcriptional regulator [*Olsenella* sp. KH3B4] |  |
|  |  | SES92801.1 | | autoinducer 2 (AI-2) kinase [*Olsenella* sp. KH3B4] |  |
|  | *Olsenella* *umbonata* DSM 22619 |  | | N/A |  |
|  | *Olsenella* *umbonata* KHGC19 |  | | N/A |  |
|  | *Olsenella* *umbonata* WCP15 |  | | N/A |  |
|  | *Oribacterium* sp. FC2011 | WP_031548817.1 | | S-ribosylhomocysteine lyase [*Oribacterium* sp. FC2011] |  |
|  |  | WP_031552425.1 | | hypothetical protein [*Oribacterium* sp. FC2011] |  |
|  | *Oribacterium* sp. KHPX15 | SEA75922.1 | | S-ribosylhomocysteine lyase /quorum-sensing autoinducer 2 (AI-2) synthesis protein LuxS [*Oribacterium* sp. KHPX15] |  |
|  |  | SEA00946.1 | | two component transcriptional regulator, LuxR family [*Oribacterium* sp. KHPX15] |  |
|  | *Oribacterium* sp. NK2B42 | WP_029199621.1 | | S-ribosylhomocysteine lyase [*Oribacterium* sp. NK2B42] |  |
|  |  | WP_029201905.1 | | LuxR family transcriptional regulator [*Oribacterium* sp. NK2B42] |  |
|  | *Oribacterium* sp. P6A1 | WP_036606466.1 | | S-ribosylhomocysteine lyase [*Oribacterium* sp. P6A1] |  |
|  |  | WP_036611134.1 | | LuxR family transcriptional regulator [*Oribacterium* sp. P6A1] |  |
|  | *Oribacterium* sp. WCC10 | SFG21674.1 | | S-ribosylhomocysteine lyase /quorum-sensing autoinducer 2 (AI-2) synthesis protein LuxS [*Oribacterium* sp. WCC10] |  |
|  |  | WP_091208409.1 | | LuxR family transcriptional regulator [*Oribacterium* sp. WCC10] |  |
|  | *Pediococcus acidilactici* AGR20 |  | | N/A |  |
|  | *Peptostreptococcaceae* *bacterium* pGA-8 | WP_092088637.1 | | LuxR family transcriptional regulator [*Peptostreptococcaceae* *bacterium* pGA-8] |  |
|  | *Peptostreptococcaceae* *bacterium* VA2 | WP_026901628.1 | | LuxR family transcriptional regulator [*Peptostreptococcaceae* *bacterium* VA2] |  |
|  |  | WP_082209551.1 | | cyclic lactone autoinducer peptide [*Peptostreptococcaceae* *bacterium* VA2] |  |
|  | *Peptostreptococcus* *anaerobius* C |  | | N/A |  |
|  | *Peptostreptococcus russellii* Calf135 |  | | N/A |  |
|  | *Peptostreptococcus* sp. D1 | SFE69394.1 | | S-ribosylhomocysteine lyase /quorum-sensing autoinducer 2 (AI-2) synthesis protein LuxS [*Peptostreptococcus* sp. D1] |  |
|  | *Propionibacteriaceae* *bacterium* P6A17 |  | | N/A |  |
|  | *Propionibacterium acnes* NLAE-zl-G260 |  | | N/A |  |
|  | *Propionibacterium* sp. MB3007 | WP_002517247.1 | | MULTISPECIES: hypothetical protein [*Propionibacteriaceae*] |  |
|  | *Pseudoramibacter* sp. KHCV1 |  | | N/A |  |
|  | *Ruaniaceae* *bacterium* KH17 | SNU01037.1 | | S-ribosylhomocysteine lyase /quorum-sensing autoinducer 2 (AI-2) synthesis protein LuxS [*Ruaniaceae* *bacterium* KH17] |  |
|  |  | SNU00370.1 | | regulatory protein, LuxR family [*Ruaniaceae* *bacterium* KH17] |  |
|  | *Ruminococcaceae* *bacterium* AB4001 |  | | N/A |  |
|  | *Ruminococcaceae* *bacterium* AE2021 |  | | N/A |  |
|  | *Ruminococcaceae* *bacterium* D5 |  | | N/A |  |
|  | *Ruminococcaceae* *bacterium* FB2012 | SDB53682.1 | | two component transcriptional regulator, LuxR family [*Ruminococcaceae* *bacterium* FB2012] |  |
|  | *Ruminococcaceae* *bacterium* KH2T8 | SEV89061.1 | | two component transcriptional regulator, LuxR family [*Ruminococcaceae* *bacterium* KH2T8] |  |
|  |  | SEW26758.1 | | cyclic lactone autoinducer peptide [*Ruminococcaceae* *bacterium* KH2T8] |  |
|  | *Ruminococcaceae* *bacterium* KHP2 |  | | N/A |  |
|  | *Ruminococcaceae* *bacterium* P7 | SCX14238.1 | | S-ribosylhomocysteine lyase /quorum-sensing autoinducer 2 (AI-2) synthesis protein LuxS [*Ruminococcaceae* *bacterium* P7] |  |
|  | *Ruminococcaceae* *bacterium* R-25 | PWJ68428.1 | | cyclic lactone autoinducer peptide [*Ruminococcaceae* *bacterium* R-25] |  |
|  | *Ruminococcaceae* *bacterium* YAD3003 |  | | N/A |  |
|  | *Ruminococcaceae* *bacterium* YRB3002 |  | | N/A |  |
|  | *Ruminococcaceae* *bacterium* YSB2003 |  | | N/A |  |
|  | *Ruminococcus albus* | WP_024856983.1 | | S-ribosylhomocysteine lyase [*Ruminococcus albus*] |  |
|  |  | WP_024857758.1 | | DNA-binding response regulator [*Ruminococcus albus*] |  |
|  |  | WP_080678301.1 | | cyclic lactone autoinducer peptide [*Ruminococcus albus*] |  |
|  | *Ruminococcus albus* AD2013 | WP_024856983.1 | | S-ribosylhomocysteine lyase [*Ruminococcus albus*] |  |
|  |  | WP_080678301.1 | | cyclic lactone autoinducer peptide [*Ruminococcus albus*] |  |
|  | *Ruminococcus albus* AR67 |  | | N/A |  |
|  | *Ruminococcus albus* KH2T6 |  | | N/A |  |
|  | *Ruminococcus bromii* | PKD32796.1 | | S-ribosylhomocysteine lyase [*Ruminococcus bromii*] |  |
|  |  | SPE92811.1 | | Response regulator protein vraR, transcriptional regulator NarL, Response regulator of citrate/malate metabolism transcriptional regulator EpsA, Bacterial regulatory proteins LuxR family [*Ruminococcus bromii* L2-63] |  |
|  |  | SCY01884.1 | | N-acyl homoserine lactone hydrolase [*Ruminococcus bromii*] |  |
|  |  | WP_101029126.1 | | cyclic lactone autoinducer peptide [*Ruminococcus bromii*] |  |
|  |  | PKD30307.1 | | autoinducer 2 ABC transporter permease LsrC [*Ruminococcus bromii*] |  |
|  | *Ruminococcus bromii* YE282 |  | | N/A |  |
|  | *Ruminococcus champanellensis* | WP_082385969.1 | | S-ribosylhomocysteine lyase [*Ruminococcus champanellensis*] |  |
|  |  | WP_015558144.1 | | DNA-binding response regulator [*Ruminococcus champanellensis*] |  |
|  |  | WP_022127260.1 | | MULTISPECIES: cyclic lactone autoinducer peptide [*Ruminococcus*] |  |
|  | *Ruminococcus* *flavefaciens* AE3010 | WP_024861168.1 | | S-ribosylhomocysteine lyase [*Ruminococcus* *flavefaciens*] |  |
|  |  | WP_080770476.1 | | cyclic lactone autoinducer peptide [*Ruminococcus* *flavefaciens*] |  |
|  | *Ruminococcus* *flavefaciens* MA2007 | WP_028515097.1 | | S-ribosylhomocysteine lyase [*Ruminococcus* *flavefaciens*] |  |
|  |  | WP_080693324.1 | | cyclic lactone autoinducer peptide [*Ruminococcus* *flavefaciens*] |  |
|  | *Ruminococcus* *flavefaciens* MC2020 |  | | N/A |  |
|  | *Ruminococcus* *flavefaciens* ND2009 | WP_028521669.1 | | S-ribosylhomocysteine lyase [*Ruminococcus* *flavefaciens*] |  |
|  |  | WP_082320566.1 | | cyclic lactone autoinducer peptide [*Ruminococcus* *flavefaciens*] |  |
|  | *Ruminococcus* *flavefaciens* SAb67 |  | | N/A |  |
|  | *Ruminococcus* *flavefaciens* XPD3002 |  | | N/A |  |
|  | *Ruminococcus* *flavefaciens* Y1 |  | | N/A |  |
|  | *Ruminococcus* *flavefaciens* YAD2003 |  | | N/A |  |
|  | *Ruminococcus* *flavefaciens* YL228 |  | | N/A |  |
|  | *Ruminococcus* *flavefaciens* YRD2003 |  | | N/A |  |
|  | *Ruminococcus* *gnavus* AGR2154 | WP_024854548.1 | | S-ribosylhomocysteine lyase [[*Ruminococcus*] *gnavus*] |  |
|  | *Ruminococcus* sp. FC2018 | WP_028505826.1 | | S-ribosylhomocysteine lyase [*Ruminococcus* sp. FC2018] |  |
|  | *Ruminococcus* sp. HUN007 | WP_044977068.1 | | S-ribosylhomocysteine lyase [*Ruminococcus* sp. HUN007] |  |
|  |  | WP_081850151.1 | | cyclic lactone autoinducer peptide [*Ruminococcus* sp. HUN007] |  |
|  | *Ruminococcus* sp. NK3A76 | WP_084262086.1 | | cyclic lactone autoinducer peptide [*Ruminococcus* sp. NK3A76] |  |
|  | *Ruminococcus* sp. YE71 | SFW20740.1 | | two component transcriptional regulator, LuxR family [*Ruminococcus* sp. YE71] |  |
|  | *Ruminococcus* sp. YE78 | SDA14230.1 | | two component transcriptional regulator, LuxR family [*Ruminococcus* sp. YE78] |  |
|  | *Sarcina* sp. DSM 11001 | SDL28745.1 | | S-ribosylhomocysteine lyase /quorum-sensing autoinducer 2 (AI-2) synthesis protein LuxS [*Sarcina* sp. DSM 11001] |  |
|  |  | WP_092992539.1 | | LuxR family transcriptional regulator [*Sarcina* sp. DSM 11001] |  |
|  |  | WP_092992078.1 | | hypothetical protein [*Sarcina* sp. DSM 11001] |  |
|  | *Sharpea azabuensis* DSM 18934 | WP_033163660.1 | | S-ribosylhomocysteine lyase [*Sharpea azabuensis*] |  |
|  | *Sharpea azabuensis* DSM 20406 |  | | N/A |  |
|  | *Sharpea azabuensis* KH1P5 |  | | N/A |  |
|  | *Sharpea azabuensis* KH2P10 |  | | N/A |  |
|  | *Slackia heliotrinireducens* | WP_041422579.1 | | LuxR family transcriptional regulator [*Slackia heliotrinireducens*] |  |
|  |  | WP_012798977.1 | | N-acyl homoserine lactonase family protein [*Slackia heliotrinireducens*] |  |
|  | *Staphylococcus epidermidis* AG42 | WP_002468352.1 | | MULTISPECIES: cyclic lactone autoinducer peptide [*Staphylococcus*] |  |
|  | *Staphylococcus epidermidis* NLAE-zl-G239 |  | | N/A |  |
|  | *Streptococcus bovis* 2B | | | N/A |  |
|  | *Streptococcus bovis* AG46 |  | | N/A |  |
|  | *Streptococcus bovis* B315 |  | | N/A |  |
|  | *Streptococcus bovis* C277 |  | | N/A |  |
|  | *Streptococcus bovis* ES1 |  | | N/A |  |
|  | *Streptococcus bovis* JB1 | SFL24156.1 | | S-ribosylhomocysteine lyase /quorum-sensing autoinducer 2 (AI-2) synthesis protein LuxS [*Streptococcus* *equinus* JB1] |  |
|  |  | KFN85899.1 | | LuxR family transcriptional regulator [*Streptococcus* *equinus* JB1] |  |
|  | *Streptococcus bovis* SN033 |  | | N/A |  |
|  | *Streptococcus* *equinus* AR3 |  | | N/A |  |
|  | *Streptococcus* *equinus* GA-1 |  | | N/A |  |
|  | *Streptococcus* *equinus* H24 |  | | N/A |  |
|  | *Streptococcus* *equinus* MPR1 |  | | N/A |  |
|  | *Streptococcus* *equinus* MPR2 |  | | N/A |  |
|  | *Streptococcus* *equinus* MPR4 |  | | N/A |  |
|  | *Streptococcus* *equinus* pGA-7 |  | | N/A |  |
|  | *Streptococcus* *equinus* pR-5 |  | | N/A |  |
|  | *Streptococcus* *equinus* Sb04 |  | | N/A |  |
|  | *Streptococcus* *equinus* Sb05 |  | | N/A |  |
|  | *Streptococcus* *equinus* Sb09 |  | | N/A |  |
|  | *Streptococcus* *equinus* Sb10 |  | | N/A |  |
|  | *Streptococcus* *equinus* Sb13 |  | | N/A |  |
|  | *Streptococcus* *equinus* Sb17 |  | | N/A |  |
|  | *Streptococcus* *equinus* Sb18 |  | | N/A |  |
|  | *Streptococcus* *equinus* Sb20 |  | | N/A |  |
|  | *Streptococcus* *equinus* SI |  | | N/A |  |
|  | *Streptococcus* *equinus* Ye01 |  | | N/A |  |
|  | *Streptococcus* *gallolyticus* LMG 15572 |  | | N/A |  |
|  | *Streptococcus* *gallolyticus* VTM1R27 |  | | N/A |  |
|  | *Streptococcus* *gallolyticus* VTM1R29 |  | | N/A |  |
|  | *Streptococcus* *gallolyticus* VTM1R48 |  | | N/A |  |
|  | *Streptococcus* *gallolyticus* VTM2R47 |  | | N/A |  |
|  | *Streptococcus* *gallolyticus* VTM3R23 |  | | N/A |  |
|  | *Streptococcus* *gallolyticus* VTM3R24 |  | | N/A |  |
|  | *Streptococcus* *gallolyticus* VTM3R42 |  | | N/A |  |
|  | *Streptococcus* *henryi* A-4 |  | | N/A |  |
|  | *Streptococcus* sp. 45 | SEI86181.1 | | S-ribosylhomocysteine lyase /quorum-sensing autoinducer 2 (AI-2) synthesis protein LuxS [*Streptococcus* sp. 45] |  |
|  |  | SEI48368.1 | | two component transcriptional regulator, LuxR family [*Streptococcus* sp. 45] |  |
|  |  | SEI94357.1 | | Threonine/homoserine/homoserine lactone efflux protein [*Streptococcus* sp. 45] |  |
|  | *Streptococcus* sp. NLAE-zl-C503 | SDP58316.1 | | S-ribosylhomocysteine lyase /quorum-sensing autoinducer 2 (AI-2) synthesis protein LuxS [*Streptococcus* sp. NLAE-zl-C503] |  |
|  |  | SDP20645.1 | | two component transcriptional regulator, LuxR family [*Streptococcus* sp. NLAE-zl-C503] |  |
| **Gram-** | *Acidaminococcus fermentans* pGA-4 |  | | N/A |  |
|  | *Acidaminococcus* *fermentens* WCC6 |  | | N/A |  |
|  | *Acinetobacter* sp DSM 11652 | SEL28367.1 | | Threonine/homoserine/homoserine lactone efflux protein [*Acinetobacter* sp. DSM 11652] |  |
|  |  | SEL56134.1 | | regulatory protein, LuxR family [*Acinetobacter* sp. DSM 11652] |  |
|  | *ActinoBacillus succinogenes* | A6VM61.1 | | S-ribosylhomocysteine lyase |  |
|  |  | ABR73692.1 | | regulatory protein LuxR [*ActinoBacillus succinogenes* 130Z] |  |
|  | *Allisonella histaminiformans* DSM 15230 |  | | N/A |  |
|  | *Anaerovibrio lipolyticus* LB2005 |  | | N/A |  |
|  | *Anaerovibrio* sp. RM50 | WP_084276914.1 | | LuxR family transcriptional regulator [*Anaerovibrio* sp. RM50] |  |
|  |  | WP_084276920.1 | | hypothetical protein [*Anaerovibrio* sp. RM50] |  |
|  | *Bacteroidales* *bacterium* KHT7 | SDF00773.1 | | S-ribosylhomocysteine lyase /quorum-sensing autoinducer 2 (AI-2) synthesis protein LuxS [*Bacteroidales* *bacterium* KHT7] |  |
|  |  | SDG66398.1 | | regulatory protein, LuxR family [*Bacteroidales* *bacterium* KHT7] |  |
|  | *Bacteroidales* *bacterium* WCE2004 | SKC50898.1 | | S-ribosylhomocysteine lyase /quorum-sensing autoinducer 2 (AI-2) synthesis protein LuxS [*Bacteroidales* *bacterium* WCE2004] |  |
|  |  | WP_079593230.1 | | LuxR family transcriptional regulator [*Bacteroidales* *bacterium* WCE2004] |  |
|  | *Bacteroidales* *bacterium* WCE2008 |  | | N/A |  |
|  | *Bacteroides ovatus* NLAE-zl-C57 |  | | N/A |  |
|  | *Bacteroides* *ovatus* NLAE-zl-C500 |  | | N/A |  |
|  | *Bacteroides* sp. AR20 | WP_090614105.1 | | LuxR family transcriptional regulator [*Bacteroides*] |  |
|  | *Bacteroides* sp. AR29 | SOB99909.1 | | S-ribosylhomocysteine lyase /quorum-sensing autoinducer 2 (AI-2) synthesis protein LuxS [*Bacteroides* sp. AR29] |  |
|  |  | SOC00596.1 | | two component transcriptional regulator, LuxR family [*Bacteroides* sp. AR29] |  |
|  | *Bacteroides* sp. Ga6A1 | WP_034538973.1 | | MULTISPECIES: S-ribosylhomocysteine lyase [*Bacteroides*] |  |
|  |  | WP_051656154.1 | | MULTISPECIES: LuxR family transcriptional regulator [*Bacteroides*] |  |
|  | *Bacteroides* sp. Ga6A2 | WP_034538973.1 | | MULTISPECIES: S-ribosylhomocysteine lyase [*Bacteroides*] |  |
|  |  | WP_051656154.1 | | MULTISPECIES: LuxR family transcriptional regulator [*Bacteroides*] |  |
|  | *Bacteroides* *thetaiotaomicron* KPPR-3 |  | | N/A |  |
|  | *Bacteroides* *thetaiotaomicron* NLAE-zl-C579 | N/A | |  |  |
|  | *Bacteroides* *vulgatus* NLAE-zl-G202 | | |  | N/A |
|  | *Bacteroides* *xylanisolvens* NLAE-zl-C202 |  | | N/A |  |
|  | *Bacteroides* *xylanisolvens* NLAE-zl-G339 |  | | N/A |  |
|  | *Basfia succiniciproducens* DSM 22022 |  | | N/A |  |
|  | *Basfia succiniciproducens* KPR-2 |  | | N/A |  |
|  | *Citrobacter* sp. NLAE-zl-C269 | SFA74219.1 | | S-ribosylhomocysteine lyase /quorum-sensing autoinducer 2 (AI-2) synthesis protein LuxS [*Citrobacter* sp. NLAE-zl-C269] |  |
|  |  | SFA71648.1 | | regulatory protein, LuxR family [*Citrobacter* sp. NLAE-zl-C269] |  |
|  |  | SFB23757.1 | | monosaccharide ABC transporter substrate-binding protein, CUT2 family [*Citrobacter* sp. NLAE-zl-C269] |  |
|  |  | SFA71754.1 | | acyl homoserine lactone synthase [*Citrobacter* sp. NLAE-zl-C269] |  |
|  | *Desulfovibrio desulfuricans* DSM 7057 |  | | N/A |  |
|  | *Desulfovibrio legallii* KHC7 |  | | N/A |  |
|  | *Enterobacter* sp. KPR-6 | SFR16536.1 | | S-ribosylhomocysteine lyase /quorum-sensing autoinducer 2 (AI-2) synthesis protein LuxS [*Enterobacter* sp. kpr-6] |  |
|  |  | SFQ95725.1 | | two component transcriptional regulator, LuxR family [*Enterobacter* sp. kpr-6] |  |
|  |  | WP_090463650.1 | | autoinducer 2 ABC transporter substrate-binding protein LsrB [*Enterobacter* sp. kpr-6] |  |
|  |  | WP_090463658.1 | | autoinducer 2 ABC transporter ATP-binding protein LsrA [*Enterobacter* sp. kpr-6] |  |
|  |  | WP_090463653.1 | | autoinducer 2 import system permease LsrD [*Enterobacter* sp. kpr-6] |  |
|  |  | SFR14032.1 | | autoinducer 2-degrading protein [*Enterobacter* sp. kpr-6] |  |
|  |  | SFR14056.1 | | autoinducer 2 (AI-2) kinase [*Enterobacter* sp. kpr-6] |  |
|  |  | WP_090465486.1 | | homoserine/homoserine lactone efflux protein [*Enterobacter* sp. kpr-6] |  |
|  | *Escherichia coli* PA-3 | EIN52330.1 | | S-ribosylhomocysteine lyase [*Escherichia coli* PA3] |  |
|  |  | WP_001303994.1 | | LuxR family transcriptional regulator [*Escherichia coli*] |  |
|  |  | WP_000981355.1 | | autoinducer 2 ABC transporter substrate-binding protein [*Escherichia coli*] |  |
|  |  | WP_001222737.1 | | autoinducer 2 import system permease LsrD [*Escherichia coli*] |  |
|  |  | WP_000911140.1 | | autoinducer 2 import system permease LsrC [*Escherichia coli*] |  |
|  |  | WP_001194917.1 | | autoinducer 2 import ATP-binding protein LsrA [*Escherichia coli*] |  |
|  |  | EIN59402.1 | | autoinducer 2-degrading protein lsrG [*Escherichia coli* PA3] |  |
|  |  | EIN59400.1 | | autoinducer 2-binding protein lsrB [*Escherichia coli* PA3] |  |
|  |  | EIN59399.1 | | autoinducer 2 import system permease protein lsrD [*Escherichia coli* PA3] |  |
|  |  | EIN59395.1 | | autoinducer 2 kinase LsrK [*Escherichia coli* PA3] |  |
|  |  | EIN57515.1 | | AI-2 transport protein tqsA [*Escherichia coli* PA3] |  |
|  | *Fibrobacter succinogenes* | ADL26680.1 | | transcriptional regulator, LuxR family [*Fibrobacter succinogenes* subsp. *succinogenes* S85] |  |
|  | *Fibrobacter* *succinogenes* HM2 |  | | N/A |  |
|  | *Fibrobacter* *succinogenes* LB2010 |  | | N/A |  |
|  | *Fusobacterium* *necrophorum* HUN048 | WP_005957266.1 | | LuxR family transcriptional regulator [Fuso*bacterium* *necrophorum*] |  |
|  | *Megamonas* sp. Calf98-2 | SEN70492.1 | | regulatory protein, LuxR family [*Megamonas* sp. Calf98-2] |  |
|  | *Megasphaera elsdenii* J1 |  | | N/A |  |
|  | *Megasphaera elsdenii* T81 |  | | N/A |  |
|  | *Megasphaera elsdenii* YE34 |  | | N/A |  |
|  | *Mitsuokella jalaludinii* DSM 13811 |  | | N/A |  |
|  | *Morganella morganii* NLAE-zl-C84 |  | | N/A |  |
|  | *Oscillibacter* sp. PC13 | SFP70447.1 | | S-ribosylhomocysteine lyase /quorum-sensing autoinducer 2 (AI-2) synthesis protein LuxS [*Oscillibacter* sp. PC13] |  |
|  |  | WP_091132885.1 | | LuxR family transcriptional regulator [*Oscillibacter* sp. PC13] |  |
|  |  | WP_091129995.1 | | N-acyl homoserine lactonase family protein [*Oscillibacter* sp. PC13] |  |
|  | *Oxalobacter formigenes* | WP_036602645.1 | | LuxR family transcriptional regulator [*Oxalobacter formigenes*] |  |
|  |  | ARQ45738.1 | | N-acyl homoserine lactonase [*Oxalobacter formigenes*] |  |
|  |  | ARQ46548.1 | | Transcriptional regulatory protein UhpA [*Oxalobacter formigenes*] |  |
|  |  | ARQ46605.1 | | Autoinducer 2 sensor kinase/phosphatase LuxQ [*Oxalobacter formigenes*] |  |
|  |  | ARQ46979.1 | | Homoserine/homoserine lactone efflux protein [*Oxalobacter formigenes*] |  |
|  |  | ARQ79012.1 | | threonine transporter RhtB [*Oxalobacter formigenes* OXCC13] |  |
|  | *Porphyromonadaceae* *bacterium* KH3CP3RA | SFK98873.1 | | regulatory protein, LuxR family [*Porphyromonadaceae* *bacterium* KH3CP3RA] |  |
|  | *Porphyromonadaceae* *bacterium* KH3R12 | SEA53547.1 | | regulatory protein, LuxR family [*Porphyromonadaceae* *bacterium* KH3R12] |  |
|  |  | SEA49463.1 | | two component transcriptional regulator, LuxR family [*Porphyromonadaceae* *bacterium* KH3R12] |  |
|  | *Porphyromonadaceae* *bacterium* KHP3R9 | SFU49018.1 | | regulatory protein, LuxR family [*Porphyromonadaceae* *bacterium* KHP3R9] |  |
|  | *Porphyromonadaceae* *bacterium* NLAE-zl-C104 | SFS51947.1 | | regulatory protein, LuxR family [*Porphyromonadaceae* *bacterium* NLAE-zl-C104] |  |
|  |  | SFS47930.1 | | Threonine/homoserine/homoserine lactone efflux protein [*Porphyromonadaceae* *bacterium* NLAE-zl-C104] |  |
|  |  | SFS49006.1 | | two component transcriptional regulator, LuxR family [*Porphyromonadaceae* *bacterium* NLAE-zl-C104] |  |
|  | *Prevotella albensis* KHC3 |  | | N/A |  |
|  | *Prevotella* *brevis* P6B11 |  | | N/A |  |
|  | *Prevotella* *bryantii* C21a | WP_081657147.1 | | LuxR family transcriptional regulator [*Prevotella* *bryantii*] |  |
|  | *Prevotella* *bryantii* FB3001 |  | | N/A |  |
|  | *Prevotella* *bryantii* KHPX14 |  | | N/A |  |
|  | *Prevotella ruminicola* | SHK82531.1 | | S-ribosylhomocysteine lyase /quorum-sensing autoinducer 2 (AI-2) synthesis protein LuxS [*Prevotella ruminicola*] |  |
|  |  | WP_103916188.1 | | LuxR family transcriptional regulator [*Prevotella ruminicola*] |  |
|  | *Prevotella ruminicola* 223/M2/7 |  | | N/A |  |
|  | *Prevotella ruminicola* AR32 |  | | N/A |  |
|  | *Prevotella ruminicola* BPI-34 |  | | N/A |  |
|  | *Prevotella ruminicola* BPI-162 |  | | N/A |  |
|  | *Prevotella ruminicola* D31d |  | | N/A |  |
|  | *Prevotella ruminicola* Ga6B6 |  | | N/A |  |
|  | *Prevotella ruminicola* KHT3 |  | | N/A |  |
|  | *Prevotella* sp. AGR2160 | WP_028909709.1 | | S-ribosylhomocysteine lyase [*Prevotella* sp. AGR2160] |  |
|  | *Prevotella* sp. BP1-145 | SDO29470.1 | | S-ribosylhomocysteine lyase /quorum-sensing autoinducer 2 (AI-2) synthesis protein LuxS [*Prevotella* sp. BP1-145] |  |
|  |  | WP_091853617.1 | | LuxR family transcriptional regulator [*Prevotella* sp. BP1-145] |  |
|  | *Prevotella* sp. BP1-148 | SDH23231.1 | | S-ribosylhomocysteine lyase /quorum-sensing autoinducer 2 (AI-2) synthesis protein LuxS [*Prevotella* sp. BP1-148] |  |
|  |  | WP_091816630.1 | | Motility quorum-sensing regulator (MqsR) [*Prevotella* sp. BP1-148] |  |
|  | *Prevotella* sp. FD3004 |  | | N/A |  |
|  | *Prevotella* sp. HUN102 | WP_028896512.1 | | S-ribosylhomocysteine lyase [*Prevotella* sp. HUN102] |  |
|  |  | WP_028898452.1 | | LuxR family transcriptional regulator [*Prevotella* sp. HUN102] |  |
|  | *Prevotella* sp. KH1P2 | SES75666.1 | | S-ribosylhomocysteine lyase /quorum-sensing autoinducer 2 (AI-2) synthesis protein LuxS [*Prevotella* sp. kh1p2] |  |
|  |  | WP_091819835.1 | | LuxR family transcriptional regulator [*Prevotella* sp. kh1p2] |  |
|  |  | SET10591.1 | | Threonine/homoserine/homoserine lactone efflux protein [*Prevotella* sp. kh1p2] |  |
|  | *Prevotella* sp. KH2C16 | SFG41830.1 | | S-ribosylhomocysteine lyase /quorum-sensing autoinducer 2 (AI-2) synthesis protein LuxS [*Prevotella* sp. KH2C16] |  |
|  |  | WP_092113427.1 | | LuxR family transcriptional regulator [*Prevotella* sp. KH2C16] |  |
|  | *Prevotella* sp. KHC14 |  | | N/A |  |
|  | *Prevotella* sp. KHP1 | SDQ75998.1 | | S-ribosylhomocysteine lyase /quorum-sensing autoinducer 2 (AI-2) synthesis protein LuxS [*Prevotella* sp. khp1] |  |
|  | *Prevotella* sp. KHP7 | SEW10453.1 | | S-ribosylhomocysteine lyase /quorum-sensing autoinducer 2 (AI-2) synthesis protein LuxS [*Prevotella* sp. khp7] |  |
|  |  | SEW03473.1 | | regulatory protein, LuxR family [*Prevotella* sp. khp7] |  |
|  | *Prevotella* sp. LC2012 | SEE51366.1 | | S-ribosylhomocysteine lyase /quorum-sensing autoinducer 2 (AI-2) synthesis protein LuxS [*Prevotella* sp. lc2012] |  |
|  |  | SEE14436.1 | | regulatory protein, LuxR family [*Prevotella* sp. lc2012] |  |
|  | *Prevotella* sp. MA2016 |  | | N/A |  |
|  | *Prevotella* sp. NE3005 | SEN12321.1 | | S-ribosylhomocysteine lyase /quorum-sensing autoinducer 2 (AI-2) synthesis protein LuxS [*Prevotella* sp. ne3005] |  |
|  |  | SEN35415.1 | | regulatory protein, luxR family [*Prevotella* sp. ne3005] |  |
|  | *Prevotella* sp. P6B1 |  | | N/A |  |
|  | *Prevotella* sp. P6B4 |  | | N/A |  |
|  | *Prevotella* sp. RM4 |  | | N/A |  |
|  | *Prevotella* sp. TC2-24 | SEW05678.1 | | S-ribosylhomocysteine lyase /quorum-sensing autoinducer 2 (AI-2) synthesis protein LuxS [*Prevotella* aff. *ruminicola* Tc2-24] |  |
|  |  | SEV88988.1 | | regulatory protein, LuxR family [*Prevotella* aff. *ruminicola* Tc2-24] |  |
|  | *Prevotella* sp. TC2-28 | SDZ94912.1 | | S-ribosylhomocysteine lyase /quorum-sensing autoinducer 2 (AI-2) synthesis protein LuxS [*Prevotella* sp. tc2-28] |  |
|  |  | SEA62392.1 | | regulatory protein, LuxR family [*Prevotella* sp. tc2-28] |  |
|  | *Prevotella* sp. TF2-5 | SFO50711.1 | | S-ribosylhomocysteine lyase /quorum-sensing autoinducer 2 (AI-2) synthesis protein LuxS [*Prevotella* sp. tf2-5] |  |
|  |  | WP_092069282.1 | | LuxR family transcriptional regulator [*Prevotella* sp. tf2-5] |  |
|  | *Prevotellaceae bacterium* HUN156 | SFW27576.1 | | S-ribosylhomocysteine lyase /quorum-sensing autoinducer 2 (AI-2) synthesis protein LuxS [*Prevotellaceae* *bacterium* HUN156] |  |
|  |  | WP_083429310.1 | | LuxR family transcriptional regulator [*Prevotellaceae* *bacterium* HUN156] |  |
|  | *Prevotellaceae* *bacterium* KH2P17 | SNU10510.1 | | S-ribosylhomocysteine lyase /quorum-sensing autoinducer 2 (AI-2) synthesis protein LuxS [*Prevotellaceae* *bacterium* KH2P17] |  |
|  |  | WP_091819835.1 | | LuxR family transcriptional regulator [*Prevotella* sp. kh1p2] |  |
|  | *Prevotellaceae* *bacterium* MN60 | SNU03761.1 | | S-ribosylhomocysteine lyase /quorum-sensing autoinducer 2 (AI-2) synthesis protein LuxS [*Prevotellaceae* *bacterium* MN60] |  |
|  | *Proteiniclasticum* *ruminis* DSM 24773 | WP_031576669.1 | | LuxR family transcriptional regulator [*Proteiniclasticum* *ruminis*] |  |
|  |  | WP_036909555.1 | | cyclic lactone autoinducer peptide [*Proteiniclasticum* *ruminis*] |  |
|  | *Proteus mirabilis* NLAE-zl-C285 |  | | N/A |  |
|  | *Proteus mirabilis* NLAE-zl-G534 |  | | N/A |  |
|  | *Pseudobutyrivibrio* *ruminis* ACV-9 |  | | N/A |  |
|  | *Pseudobutyrivibrio* *ruminis* AD2017 | WP_028243313.1 | | S-ribosylhomocysteine lyase [*Pseudobutyrivibrio* *ruminis*] |  |
|  | *Pseudobutyrivibrio* *ruminis* CF1b | WP_028247633.1 | | MULTISPECIES: S-ribosylhomocysteine lyase [*Pseudobutyrivibrio*] |  |
|  | *Pseudobutyrivibrio* *ruminis* DSM 9787 | SOB98252.1 | | S-ribosylhomocysteine lyase /quorum-sensing autoinducer 2 (AI-2) synthesis protein LuxS [*Pseudobutyrivibrio* *ruminis* DSM 9787] |  |
|  | *Pseudobutyrivibrio* *ruminis* HUN009 | WP_033152897.1 | | S-ribosylhomocysteine lyase [*Pseudobutyrivibrio* *ruminis*] |  |
|  |  | WP_033151583.1 | | LuxR family transcriptional regulator [*Pseudobutyrivibrio* *ruminis*] |  |
|  | *Pseudobutyrivibrio* sp. 49 | SDH87805.1 | | S-ribosylhomocysteine lyase /quorum-sensing autoinducer 2 (AI-2) synthesis protein LuxS [*Pseudobutyrivibrio* sp. 49] |  |
|  |  | SDI28932.1 | | regulatory protein, LuxR family [*Pseudobutyrivibrio* sp. 49] |  |
|  |  | WP_090170118.1 | | cyclic lactone autoinducer peptide [*Pseudobutyrivibrio* sp. 49] |  |
|  | *Pseudobutyrivibrio* sp. ACV-2 | SEA77748.1 | | S-ribosylhomocysteine lyase /quorum-sensing autoinducer 2 (AI-2) synthesis protein LuxS [*Pseudobutyrivibrio* sp. ACV-2] |  |
|  |  | SEB07662.1 | | cyclic lactone autoinducer peptide [*Pseudobutyrivibrio* sp. ACV-2] |  |
|  | *Pseudobutyrivibrio* sp AR14 | SCY43135.1 | | S-ribosylhomocysteine lyase /quorum-sensing autoinducer 2 (AI-2) synthesis protein LuxS [*Pseudobutyrivibrio* sp. AR14] |  |
|  |  | WP_090153078.1 | | cyclic lactone autoinducer peptide [*Pseudobutyrivibrio* sp. AR14] |  |
|  | *Pseudobutyrivibrio* sp. AR73 |  | | N/A |  |
|  | *Pseudobutyrivibrio* sp. C4 | SET10939.1 | | S-ribosylhomocysteine lyase /quorum-sensing autoinducer 2 (AI-2) synthesis protein LuxS [*Pseudobutyrivibrio* sp. C4] |  |
|  | *Pseudobutyrivibrio* sp. CF3 |  | | N/A |  |
|  | *Pseudobutyrivibrio* sp. GS111 |  | | N/A |  |
|  | *Pseudobutyrivibrio* sp. JW11 | SFO54566.1 | | S-ribosylhomocysteine lyase /quorum-sensing autoinducer 2 (AI-2) synthesis protein LuxS [*Pseudobutyrivibrio* sp. JW11] |  |
|  |  | WP_090478510.1 | | LuxR family transcriptional regulator [*Pseudobutyrivibrio* sp. JW11] |  |
|  | *Pseudobutyrivibrio* sp. LB2011 | WP_044936347.1 | | MULTISPECIES: S-ribosylhomocysteine lyase [*Pseudobutyrivibrio*] |  |
|  | *Pseudobutyrivibrio* sp. M3.8 |  | | N/A |  |
|  | *Pseudobutyrivibrio* sp. MD2005 | WP_028234506.1 | | S-ribosylhomocysteine lyase [*Pseudobutyrivibrio* sp. MD2005] |  |
|  | *Pseudobutyrivibrio* sp. NOR37 | SFR71364.1 | | S-ribosylhomocysteine lyase /quorum-sensing autoinducer 2 (AI-2) synthesis protein LuxS [*Pseudobutyrivibrio* sp. NOR37] |  |
|  |  | WP_090488194.1 | | cyclic lactone autoinducer peptide [*Pseudobutyrivibrio* sp. NOR37] |  |
|  | *Pseudobutyrivibrio* sp. OR37 | SFI11795.1 | | S-ribosylhomocysteine lyase /quorum-sensing autoinducer 2 (AI-2) synthesis protein LuxS [*Pseudobutyrivibrio* sp. OR37] |  |
|  | *Pseudobutyrivibrio* sp. UC1225 | SFO18439.1 | | S-ribosylhomocysteine lyase /quorum-sensing autoinducer 2 (AI-2) synthesis protein LuxS [*Pseudobutyrivibrio* sp. UC1225] |  |
|  |  | SFN84871.1 | | regulatory protein, LuxR family [*Pseudobutyrivibrio* sp. UC1225] |  |
|  |  | WP_090529190.1 | | cyclic lactone autoinducer peptide [*Pseudobutyrivibrio* sp. UC1225] |  |
|  | *Pseudobutyrivibrio* sp. VV1 |  | | N/A |  |
|  | *Pseudobutyrivibrio* sp. YE44 | SDB30660.1 | | S-ribosylhomocysteine lyase /quorum-sensing autoinducer 2 (AI-2) synthesis protein LuxS [*Pseudobutyrivibrio* sp. YE44] |  |
|  | *Pseudobutyrivibrio* *xylanivorans* DSM 10317 | N/A | |  |  |
|  | *Ruminobacter amylophilus* DSM 1361 | | |  | N/A |
|  | *Ruminobacter* sp. RM87 | WP_031579621.1 | | S-ribosylhomocysteine lyase [*Ruminobacter* sp. RM87] |  |
|  |  | WP_031580454.1 | | MULTISPECIES: UvrY/SirA/GacA family response regulator transcription factor [*Ruminobacter*] |  |
|  | *Sagittula stellata* | WP_005863035.1 | | autoinducer 2 ABC transporter substrate-binding protein [*Sagittula stellata*] |  |
|  |  | WP_005856280.1 | | LuxR family transcriptional regulator [*Sagittula stellata*] |  |
|  |  | WP_005858901.1 | | autoinducer synthase [*Sagittula stellata*] |  |
|  |  | WP_005860409.1 | | N-acyl homoserine lactonase family protein [*Sagittula stellata*] |  |
|  |  | EBA08099.1 | | autoinducer synthesis protein [*Sagittula stellata* E-37] |  |
|  |  | EBA07422.1 | | hypothetical protein SSE37_21525 [*Sagittula stellata* E-37] |  |
|  | *Selenomonas bovis* 8-14-1 |  | | N/A |  |
|  | *Selenomonas ruminantium* | WP_026759796.1 | | LuxR family transcriptional regulator [*Selenomonas ruminantium*] |  |
|  |  | WP_026765422.1 | | N-acyl homoserine lactonase family protein [*Selenomonas ruminantium*] |  |
|  | *Selenomonas* *ruminantium* AB3002 |  | | N/A |  |
|  | *Selenomonas* *ruminantium* AC2024 | WP_026759796.1 | | LuxR family transcriptional regulator [*Selenomonas* *ruminantium*] |  |
|  | *Selenomonas* *ruminantium* ATCC 12561 |  | | N/A |  |
|  | *Selenomonas* *ruminantium* C3 |  | | N/A |  |
|  | *Selenomonas* *ruminantium* GACV-9 |  | | N/A |  |
|  | *Selenomonas* *ruminantium* HD4 |  | | N/A |  |
|  | *Selenomonas* *ruminantium* KH1T6 |  | | N/A |  |
|  | *Selenomonas* *ruminantium* L14 |  | | N/A |  |
|  | *Selenomonas* *ruminantium* WCT3 |  | | N/A |  |
|  | *Selenomonas* *ruminantium* Z108 |  | | N/A |  |
|  | *Selenomonas* *ruminantium* subsp. *lactilytica* DSM 2872 |  | | N/A |  |
|  | *Selenomonas* *ruminatium* S137 |  | | N/A |  |
|  | *Selenomonas* sp. AE3005 | WP_028129992.1 | | LuxR family transcriptional regulator [*Selenomonas* sp. AE3005] |  |
|  |  | WP_080695478.1 | | MULTISPECIES: LuxR family transcriptional regulator [*Selenomonas*] |  |
|  | *Selenomonas* sp. FC4001 |  | | N/A |  |
|  | *Selenomonas* sp. ND2010 |  | | N/A |  |
|  | *Shigella* *sonnei* NLAE-zl-G496 |  | | N/A |  |
|  | *Succiniclasticum* *ruminis* DSM 9236 | WP_093913003.1 | | LuxR family transcriptional regulator [*Succiniclasticum* *ruminis*] |  |
|  | *Succiniclasticum* *ruminis* DSM11005 |  | | N/A |  |
|  | *Succinivibrio dextrinosolvens* 22B |  | | N/A |  |
|  | *Succinivibrio dextrinosolvens* ACV-10 |  | | N/A |  |
|  | *Succinivibrio dextrinosolvens* H5 | WP_031492035.1 | | autoinducer-2 kinase [*Succinivibrio dextrinosolvens*] |  |
|  | *Treponema* *bryantii* B25 |  | | N/A |  |
|  | *Treponema* *bryantii* NK4A124 | WP_022932827.1 | | Motility quorum-sensing regulator (MqsR) [*Treponema* *bryantii*] |  |
|  | *Treponema* *bryantii* XBD1002 |  | | N/A |  |
|  | *Treponema* *saccharophilum* | EIC03173.1 | | quorum-sensing autoinducer 2 (AI-2), LuxS [*Treponema* *saccharophilum* DSM 2985] |  |
|  |  | WP_002702000.1 | | LuxR family transcriptional regulator [*Treponema* *saccharophilum*] |  |
|  |  | EIC03173.1 | | quorum-sensing autoinducer 2 (AI-2), LuxS [*Treponema* *saccharophilum* DSM 2985] |  |
|  |  | EIC00572.1 | | carbon storage regulator, CsrA [*Treponema* *saccharophilum* DSM 2985] |  |
|  | *Treponema* sp. C6A8 | WP_027728926.1 | | LuxR family transcriptional regulator [*Treponema* sp. C6A8] |  |
|  | *Wolinella* *succinogenes* | Q7MQP3.1 | | S-ribosylhomocysteine lyase |  |
|  |  | CAE10838.1 | | TRANSPORTER TRANSMEMBRANE PROTEIN-Putative threonine/homoserine/homoserine lactone efflux protein [*Wolinella* *succinogenes*] |  |
|  |  | CBV32453.1 | | unnamed protein product [*Wolinella* *succinogenes*] |  |
| **variable** | *Flavonifractor plautii* P3 |  | | N/A |  |

N/A: no data found,

Reclassification: *Clostridium mangenotii* as *Clostridioides mangenotii*, *Eubacterium rectalis* as *Agathobacter rectalis*

Family: *Crodiobacteriaceae* Genus: *Olsenella*

*Propionibacteriaceae* *Propionibacterium*

*Streptococcus equinus* JB1 is the organism name of *Strepococcus bovis* JB1

S-ribosylhomocysteine lyase: synonym AI-2 synthase protein (LuxS)
